# The metabolic response of *P. putida *KT2442 producing high levels of polyhydroxyalkanoate under single- and multiple-nutrient-limited growth: Highlights from a multi-level omics approach

**DOI:** 10.1186/1475-2859-11-34

**Published:** 2012-03-20

**Authors:** Ignacio Poblete-Castro, Isabel F Escapa, Christian Jäger, Jacek Puchalka, Carolyn Ming Chi Lam, Dietmar Schomburg, María Auxiliadora Prieto, Vítor AP Martins dos Santos

**Affiliations:** 1 Systems and Synthetic Biology Group, Helmholtz Centre for Infection Research (HZI), Inhoffenstraße 7, 38124 Braunschweig, Germany; 2 Department of Environmental Biology, Centro de Investigaciones Biológicas, CSIC, c/Ramiro de Maeztu 9, 28040 Madrid, Spain; 3 Department of Bioinformatics and Biochemistry, Technische Universität Braunschweig, Langer Kamp 19B, 38106 Braunschweig, Germany; 4 Systems and Synthetic Biology, Wageningen University, Dreijenplein 10, Building number 316, 6703 HB, Wageningen, The Netherlands; 5 Department of Loss Risk and Environmental Science, Universidad Tecnológica Metropolitana, Dieciocho 390, Santiago, Chile

**Keywords:** *P. putida *KT2442, Nutrient limitation, Systems biology, Polyhydroxyalkanoates

## Abstract

**Background:**

*Pseudomonas putida *KT2442 is a natural producer of polyhydroxyalkanoates (PHAs), which can substitute petroleum-based non-renewable plastics and form the basis for the production of tailor-made biopolymers. However, despite the substantial body of work on PHA production by *P. putida *strains, it is not yet clear how the bacterium re-arranges its whole metabolism when it senses the limitation of nitrogen and the excess of fatty acids as carbon source, to result in a large accumulation of PHAs within the cell. In the present study we investigated the metabolic response of KT2442 using a systems biology approach to highlight the differences between single- and multiple-nutrient-limited growth in chemostat cultures.

**Results:**

We found that 26, 62, and 81% of the cell dry weight consist of PHA under conditions of carbon, dual, and nitrogen limitation, respectively. Under nitrogen limitation a specific PHA production rate of 0.43 (g·(g·h)^-1^) was obtained. The residual biomass was not constant for dual- and strict nitrogen-limiting growth, showing a different feature in comparison to other *P. putida *strains. Dual limitation resulted in patterns of gene expression, protein level, and metabolite concentrations that substantially differ from those observed under exclusive carbon or nitrogen limitation. The most pronounced differences were found in the energy metabolism, fatty acid metabolism, as well as stress proteins and enzymes belonging to the transport system.

**Conclusion:**

This is the first study where the interrelationship between nutrient limitations and PHA synthesis has been investigated under well-controlled conditions using a system level approach. The knowledge generated will be of great assistance for the development of bioprocesses and further metabolic engineering work in this versatile organism to both enhance and diversify the industrial production of PHAs.

## Background

Microorganisms constantly face fluctuations of nutrient concentrations in their natural environments. One of the common evoked responses by bacteria is the storage of carbon and energy sources, as shown by the considerable increase in the accumulation of various compounds, such as glycogen, polyesters, and polyphosphates etc. [1]. The primary feature of these compounds is that they can be readily degraded by the cell to satisfy metabolic demands, thus ensuring its survival during famine. *Pseudomonas putida *KT2440 is a metabolically versatile bacterium [2] normally found in aerobic and semi-aerobic soil and water habitats [3], which has become an efficient cell factory for the biotechnological production of value-added compounds [4]. It synthesizes medium-chain-length polyhydroxyalkanoate (PHA) that exhibit different physical properties than those of the first discovered polyester polyhydroxybutyrate (PHB) [5,6]. PHAs can substitute petroleum-based non-renewable plastics and form the basis for the production of tailor-made biopolymers for medical applications [7], where fermentation strategies [8] and the supplied carbon sources [9] highly influence the final monomer composition of the PHA. However, despite the substantial body of work on PHA production by *P. putida *strains, it is not yet clear how the bacterium re-arranges its whole metabolism when it senses the limitation of an inorganic (N, S, P, or O) nutrient and the excess of fatty acids as carbon source, resulting in a large accumulation of PHAs within the cell. Recently, we demonstrated that this pathway acts as an important energy and carbon buffer under nutrient-limiting conditions that guarantee efficient growth [10]. Inactivation of the pathway for PHA accumulation under low nitrogen growth conditions resulted in oxidation of the excess carbon source, rather than transforming it into biomass or secretable compounds, which could be further reused as carbon or energy sources [11]. Chemostat operation allows the single limitation of carbon or of any desirable nutrient in the culture. It is as well the best alternative to perform controlled and highly reproducible cultivation for studying the phenotype of a given organism [12], especially when applying hightroughput technology to capture the transcriptome, proteome, or metabolome of the cell for a given phenotype. Using continuous cultivation, Egli and Quayle demonstrated that varying the ratio of carbon/nitrogen in the feed medium had a significant influence in the cellular and enzymatic composition on the yeast *H. polymorpha *[13]. In addition, three distinct growth regimes were recognized: namely carbon-, carbon-nitrogen-, and strict nitrogen-limiting growth. By applying those fermentation strategies, *P. putida *GPo1 (formerly known as *P. oleovorans*) was investigated for its capacity to accumulate PHAs from different carbon sources [9,14,15], proving the high metabolic flexibility of GPo1 which was reflected in part by the broad dual-limiting area between the two single-nutrient limitation (for excellent review, see [16]). One of the most interesting findings is that the dual-nutrient-limiting regime can result in the accumulation of PHA at levels comparable to those under strict nitrogen limitation [17]. As this results in less amounts of carbon used for comparable levels of PHA, this can substantially reduce the production costs.

The release of the *P. putida *KT2440 genome sequence in 2002 [2] has enabled researchers to gain deeper and broader insights into the mechanisms underlying PHA biosynthesis [18-21]. The progress in high-throughput technologies such as transcriptomics, proteomics, and metabolomics has expanded greatly the understanding of the genotype-phenotype relationships in *Pseudomonas*. As a result, several constrain-based metabolic models of this versatile organism have been developed [22-24]. These models are useful to improve the production of PHAs, especially since the metabolic responses for PHA synthesis, which takes place preferably under the limitation of several nutrients, are complex and so far not well-understood. The work herein described aims to unravel the differences between carbon-, carbon-nitrogen-, and nitrogen-limited cultures, where omic-wide measurements were integrated to interpret the resulting phenotype for each condition in terms of PHA/biomass production in *P. putida *KT2442. This can contribute to set a basis for further development of new biocatalysts and processes that can contribute to reducing the production cost, which remains the biggest obstacle for the economically viable industrial production of PHAs.

## Results and discussion

### Physiological response of *Pseudomonas putida *under nutrient-limited conditions

In order to evaluate both the capacity of *P. putida *to produce medium-chain-length polyhydroxyalkanoates (mcl-PHAs) and the global cellular responses at the transcriptome, proteome, and metabolome levels, continuous cultivations were conducted under different nutrient-limited conditions. At least three independent experiments for each nutrient limitation were performed under aerobic chemostat conditions. To achieve metabolic steady-state at a dilution rate (*D*) of 0.1 h^-1^, five to eight times the residence volume were necessary to attain constant macroscopic physiological parameters across the time. From continuous and flask-culture cultivations the maximum specific growth rate (μ_max_) on decanoate was found to be 0.53 h^-1 ^(data not shown). When *D *approaches μ_max_, the amount of PHA decreases within the cell [25]. Therefore, we imposed a low *D *(0.1 h^-1^) to obtain as much PHA as possible. Decanoate was employed as the unique carbon and energy source, whereas ammonium [NH_4_^+^] as used as the nitrogen source. We changed the (C_0_/N_0_) ratios in the feed medium by keeping the nitrogen concentration invariable and increasing the carbon quantity. We were able to establish three specific environments within the chemostat: carbon- (C), carbon-nitrogen- (dual), and strictly nitrogen- (N) limited cultures, which were confirmed by the analytical measurement of the fermentation broth (Table 1). ^1^H spectra were recorded from samples taken under all limiting fermentation conditions. In all cases there was no significant accumulation of acetate or any other soluble small molecular weight metabolite.

**Table 1:** PHA production and relative monomer composition by *P.putida *KT2442 in continuous culture at *D *= 0.1 h^-1^

Limitation	**(C**_**0**_**/N**_**0**_)*	**Y **_**X/C**_	**Y **_**X/N**_	**(C_0_/N_0_)**^**b**^	**Residual carbon**^**a**^	**Residual nitrogen**^**a**^	**CDW**^**a**^	**PHA content**^**a**^	Monomer composition (mol %)		
Borders											
	**(mol mol**^**-1**^)	(g g^-1^)	(g g^-1^)	(mol mol^-1^)	(g)	(g)	(g l^-1^)	(% CDW)	C6	C8	C10
Carbon	5.83	1.11	9.80	**10.40**	N.D.	0.112 ± 0.01	1.46 ± 0.04	25.78 ± 0.97	3.3	53.4	43.3
Carbon-Nitrogen	16.56	1.16	16.67		N.D.	N.D.	4.35 ± 0.13	61.94 ± 4.95	4.8	42.8	52.4
Nitrogen	26.97	1.01	17.74	**20.50**	1.53 ± 0.04	N.D.	4.63 ± 0.14	80.58 ± 0.24	4.3	40.2	55.5

^a ^Standard deviation (±) from at least three independent experiments

*C_0_/N_0_: Initial feeding ratio of carbon-nitrogen

*N.D*., Not detectable

^b ^According to the procedure described by Egly and Quayle (1986)

**Figure 1:**
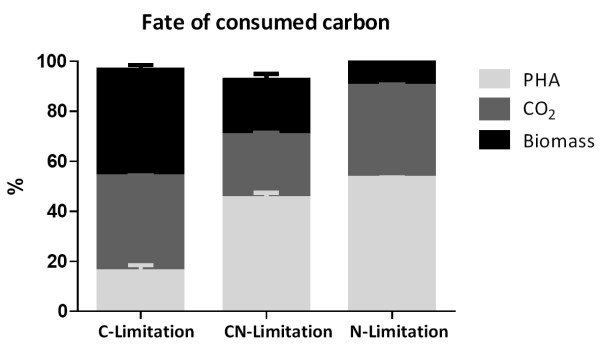
**Fate of consumed carbon based on the carbon balance within the chemostat subjected to carbon, carbon-nitrogen, and nitrogen limitation**.

**Figure 2:**
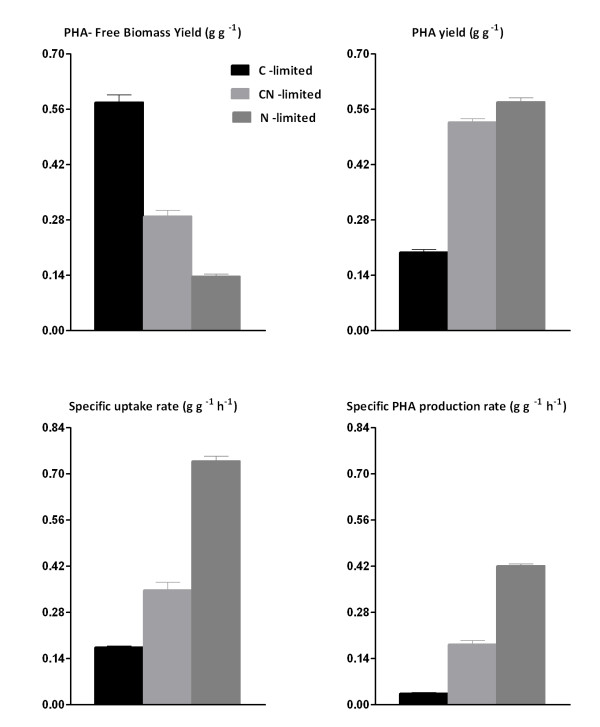
**Physiological parameters of chemostat subjected to nutrient limitations**. A) Grams of biomass (PHA-free) per gram of decanoate; B) Grams of PHA per gram of decanoate; C) Grams of decanoate per gram of biomass (PHA-free) per hour; D) Grams of PHA per gram of biomass (PHA-free) per hour.

To obtain insights into the distribution of the metabolized carbon within the cell, we have performed a carbon mass balance analysis for each condition tested (Figure 1). This was calculated based on number of moles of carbon consumed and generated within the chemostat. The C-mol content in the biomass (PHA-free) was assumed to be constant which corresponds to a molecular weight of 27 g (C-mol biomass)^-1 ^[26]. The environment which is assigned less CO_2 _(in terms of percentage) among those tested conditions is the dual limitation. A similar percentage of carbon ended up in PHAs in carbon-nitrogen- and nitrogen-deprived cells. In decanoate and decanoate-ammonium-limited cultures, mass-balances indicated that the entire carbon available in the reaction volume was converted to biomass, CO_2_, and PHA (Figure 1). As shown in (Figure 2A), the biomass yield on decanoate decreased when the ammonium concentration limited growth, expressing its major effect under strict nitrogen limitation. The physiological response of KT2442 to the nutrient-deprived environments differed in the build-up of biomass for both C-N- and N-limited cultures as compared with the C-limited culture. This might be due to the increased pool of metabolites suitable for PHA accumulation in the *β*-oxidation pathway, which consequently decreases the buffer of precursor for biomass formation, affecting the energy efficiency not only for strict N-deprived cultures [27], but also for C-N-deprived cells. The PHA yield on decanoate was very similar for both dual and strict nitrogen limitation (Figure 2B). Because of this, the additional carbon source is nearly superfluous, indicating potential for savings on the raw materials. Besides, the imposed carbon/nitrogen ratio to achieve the dual limitation environment (Table 1) was significantly higher than the calculated boundary of this condition, which is known to achieve a higher PHA yield [15]. Remarkably, *P. putida *KT2442 showed a different behavior compared to *P. putida *GPo1 with regard to the residual biomass, since for KT2442 this was not constant under CN-and N-limiting growth conditions (Table 1). For GPo1, once the growth condition reached the dual limitation area, the residual biomass remained constant while the PHA content increased proportionally with respect to the C_0_/N_0 _ratio in feed medium [14]. In our study the maximum specific PHA production rate of KT2442 was obtained when the single nitrogen limitation was imposed to the chemostat (Figure 2D), reaching a value of 0.43 (g·(g h)^-1^). This productivity is 3.5-fold higher than the one showed by GPo1 with the same dilution rate while growing on octanoate [14], and 2-fold higher than the processes using 10-undecenoate as carbon source with also *P. putida *GPo1 [9].

A) Grams of biomass (PHA-free) per gram of decanoate; B) Grams of PHA per gram of decanoate; C) Grams of decanoate per gram of biomass (PHA-free) per hour; D) Grams of PHA per gram of biomass (PHA-free) per hour

### PHA accumulation *versus *nutrient availability

To assess the capability of *P. putida *to synthesize mcl-PHAs, we used gas chromatography to quantify intracellular polyester content and characterize the monomer composition from each individual chemostat culture. The heteropolymer was synthesized under each condition, with the accumulation range of the storage compound in the cell increasing when the (C_0_/N_0_) ratio in the feed medium is higher (see Table 1). *P. putida *KT2442 was capable of synthesizing PHA up to 26% of its total cell dry weight (CDW) in carbon-limited cultures. A similar PHA concentration (20% of CDW) was found when KT2442 was grown in oleic acid with a C-mol/N-mol ratio of 10 in continuous cultures [25]. Such polymer accumulation is one of the special characteristics shown by *P. putida *strains under carbon-limiting conditions. Nevertheless, not all of them reach high levels of PHA accumulation. In the case of *P. putida *GPo1 the polyester synthesis yielded 2% of the CDW during carbon-limited growth on heptanoate and octanoate, but with nonanoate PHA accounted for 19 wt% of the biomass [15]. Under dual limitation 62% of the CDW of KT2442 was composed of PHAs, whereas under strict nitrogen-limitation this was 81%.

We were able to identify three saturated monomers in the polyester [21,28] composed of 3.3 mole% of 3-hydroxyhexanoate (C6), 53.4 mole% of 3-hydroxyoctanoate (C8), and 43.3 mole% of 3-hydrodecanoate (C10) when carbon was the limited compound. The monomer composition present in this study is extremely similar when *P. putida *KT2440 was grown in batch cultures supplemented with decanoate [28]. When decanoate is supplied as the only carbon source, the catabolic process occurs through the *β*-oxidation pathway. This correlates with the chain length structure detected in the heteropolymer showing C10, C8, and C6 hydroxyl-fatty acids as monomers, as consequence of two carbons loss during each round of the metabolic cycle. Comparing all nutrient-limited regimes, no remarkable difference could be observed in the monomer distribution. However, when ammonia was the limiting inorganic nutrient, a slight increase (of approximately 10%) in C10 was observed. When both carbon and nitrogen were bellow the detection limit in the chemostat, the monomer composition of the polymer was almost the same as the one observed in nitrogen deprived cultures (Table 1). Further discussion is made below (see fatty acid and PHA metabolism).

### Global multi-omics analysis of *P. putida *response under various nutrient limitations

#### Transcriptomics

Employing *genome-wide microarray *technology, we quantified the variation in gene expression levels of all three limiting conditions. As we set up our experiments under *steady-state*, the measurements reflected a snap-shot of the metabolic state under the desirable growth limitation by nitrogen or carbon-nitrogen. Three independent biological replicates were analyzed statistically after hybridization. The conditions of strict nitrogen limitation and carbon-nitrogen limitation were compared independently to carbon limitation, i.e. N vs. C and CN vs. C, respectively. The resulting intensities were treated using a different package from the Bioconductor suite (see material and methods). The classification of the resulting variations in gene expression were based on Cluster of Orthologous Groups (COG) (http://www.ncbi.nlm.nih.gov/COG/grace/cogenome.cgi?g=287) and illustrated in Additional file 1: Figure S1. In total, 215 genes were differentially expressed (*P *< 0.05), from which 36% of their gene products were assigned as having unknown function (Addtional file 2: Table S1). The analysis of N vs. C and CN vs. C revealed 42 and 31 upregulated genes, while those that had a fold change less than 0.5-fold were 17 and 125, respectively (Additional file 2: Table S1). As such, the N vs. C and CN vs. C comparisons shared 20 Open Reading Frames (in grey background Table 2), with 11 showing more than 2-fold differential expression (Table 2).

**Table 2:** Selected genes differentially expressed within nitrogen (N) or carbon-nitrogen (C-N) limited cultures relative to carbon limited chemostat cultures

**Cellular role category**	**Locus tag**	**Description**	**Fold change**	
			**N**	**C-N**
			
Energy metabolism				
	**PP2638**^**¥**^	**Cellulose synthase operon C putative**	**3.9**	**4.3**
	PP0813	Cytochrome *o *ubiquinol oxidase subunit I	5.0	1.2
	PP0814	Cytochrome *o *ubiquinol oxidase subunit III	4.4	-1.1
	PP4050	Glycogen synthase	3.1	1.3
	**PP0555**	**Acetoin dehydrogenase alpha subunit**	**-6.7**	**-8.3**
	**PP0556**	**Acetoin catabolism protein**	**-3.7**	**-4.6**
	**PP0554**	**Acetoin dehydrogenase beta subunit**	**-4.6**	**-4.2**
	**PP0553**	**Acetoin dehydrogenase dihydrolipoamide**	**-5.3**	**-4.0**
	**PP0557**	**Acetoin catabolism regulatory protein**	**-3.4**	**-3.6**
	PP2149	Glyceraldehyde 3-phospate dehydrogenase	1.5	-3.7
	PP4715	Triosephosphate isomerase	1.9	-3.3
	PP4737	D-lactate dehydrogenase putative	-2.6*	-3.1
	**PP0545**	**Aldehyde dehydrogenase family protein**	**-1.9**	**-3.1**
	PP1083	Bacterioferritin-associated ferredoxin putative	1.4	2.9
	PP3071	Acetoacetyl-CoA synthetase putative	1.1	-2.6
	PP4736	L- lactate dehydrogenase	-1.1	-2.5
	PP4192	Succinate dehydrogenase hydrophobic	3.9*	-2.4
	PP4193	Succinate dehydrogenase cytochrome	2.7*	-2.2
Amino acid transport and biosynthesis				
	**PP4842**	**Branched-chain amino acid ABC transport**	**15.9**	**5.8**
	**PP4841**	**Branched-chain amino acid ABC transport**	**7.3**	**4.5**
	PP5075	Glutamate synthase small subunit	3.4	1.2
	PP3021	Transporter LysE family	-1.4	2.2
Transport and binding proteins				
	PP4147	Peptide ABC transporter	-1.1	-2.7
	PP1076	Glycerol uptake facilitator protein	1.4	-2.9
	PP3954	Periplasmic binding protein putative	-1.9	-3.2
Nitrogen metabolism				
	**PP5234**	**Nitrogen regulatory protein P-II**	**25.1**	**12.2**
	**PP5233**	**Ammonium transporter**	**13.7**	**2.6**
	PP1705	Nitrate reductase	4.1*	5.2
	PP1703	Assimilatory nitrate reductase putative	2.3*	3.6
	PP2094	Nitrate-binding protein	4.5*	2.6
	PP2092	Nitrate transporter	2.3*	3.7
	PP2846	Urease accessory protein UreE	3.1*	2.8
	PP2847	Urease accessory protein UreJ	2.6*	2.5
	**PP2843**	**Urease gamma subunit UreA**	**2.3**	**2.2**
Fatty acid and phospholipid metabolism				
	PP2050	Conserved hypothetical protein	28.4	1.2
	PP2051	Acetyl-CoA acetyltransferase	27.9	-1.1
	PP2049	Alcohol dehydrogenase iron-containing	28.3	1.1
	PP2047	3-hydroxyacyl-CoA dehydrogenase family	20.9	1.1
	PP2048	Acyl-CoA dehydrogenase putative	19.8	1.5
	PP4550	Long-chain-fatty-acid-CoA ligase	4.2	1.5
Stress response				
	PP2437	Acyl-CoA dehydrogenase putative	-1.4	-3.2
	PP3781	Oxygen-independent Coproporphyrinogen III	7.5	-1.1
	PP1185	Outer membrane protein H1	6.6	2.0*
	PP1360	Chaperonin, 10 kDa	3.4	-1.1
Other functions				
	**PP2141**	**Hypothetical protein**	**-4.0**	**-3.1**
	**PP1659**	**Hypothetical protein**	**-4.8**	**-2.4**
	**PP0765**	**Hypothetical protein**	**-7.1**	**-2.8**
	**PP2685**	**Hypothetical protein**	**11.1**	**4.0**
	**PP2686**	**Transglutaminase-like superfamily**	**6.6**	**4.6**
	**PP2687**	**Conserved hypothetical protein**	**7.1**	**2.6**
	PP2689	Endoribonuclease putative	1.7	2.5

* 0.1 > *P*-value > 0.05

^¥ ^Bold font indicates matching gene expression of N vs. C and CN vs. C

#### Proteomics

Proteome profiling of *Pseudomonas putida *was performed using two-dimensional electrophoresis gels for each limiting condition to gain insight into the PHA accumulation machinery. As performed in the transcriptome analysis the gels from N- and CN-limited conditions were overlapped against the gel of C-limitation (master gel). The proteomic profile for the CN vs. C exhibited only 17 spots with differential expression (DE). A completely different scenario was detected by overlapping the nitrogen limiting growth gel with the master gel, giving 74 spots with differential expression. After searching the sequence similarity for protein identification (see materials and methods), out of these 74 spots, 45 met the requirements for protein identification. The proteins were classified using COGs and JCVI-CMR (http://cmr.jcvi.org/cgi-bin/CMR/CmrHomePage.cgi) and presented in Table 3. The transcriptome response based on DNA microarray was compared to the proteome data. For C/N vs. C limitation only 5 proteins matched with their corresponding gene expression, and for N vs. C limitation 6 fell into this category (Table 3, in grey background). Although, 2-D PAGE proteome analysis is one of the most employed techniques in the field, it did not capture the entire proteome of the tested conditions (no identification of PhaC or PhaF), thus having a significant impact on the comparison.

**Table 3:** Genes differentially expressed of the PHA metabolic pathway within nitrogen (N) or carbon-nitrogen (C-N) limited cultures relative to carbon limited chemostat cultures

**Gene name**	**Locus tag**	**Description**	**Fold change**	
			**N**	**C-N**
				
			
***phaI^¥^***	**PP5008**	**PHA granule-associated**	**6.1**	**2.5**
***phaF***	**PP5007**	**PHA granule-associated**	3.1*	**2.3**
*phaC1*	PP5003	PHA polymerase	3.4*	1.0
***phaC2***	**PP5005**	**PHA polymerase**	**1.2**	**1.1**
*phaZ*	PP5004	PHA depolymerase	2.1*	1.3
***phaD***	**PP5006**	**Transcriptional regulator**	**1.2**	**1.3**
***phaJ***	**PP4552**	**Enoyl-CoA hydratase**	**1.5**	**1.6**
***phaG***	**PP1408**	**Acyl-transferase**	3.3*	**2.2**

* 0.1 > *P*-value > 0.05

**^¥ ^**Bold font indicates matching gene expression of N vs. C and CN vs. C

### Relationships and discrepancies between transcripts and proteins

The correlation between increased transcripts and their corresponding protein abundance is very poor as shown in Table 3 for CN vs. C and N vs. C comparisons. Earlier studies have previously shown this trend between mRNA and protein levels [29-31]. It has mainly been attributed to post-transcriptional events [29] e.g. translation efficiency or protein degradation, and/or false-positive results either on the mRNA or protein levels [32]. The generated gels from the proteome analysis in this study were made in triplicate for each tested condition, giving a confident proteome profile. Therefore, the high discrepancy between the transcript and protein expression levels, for both comparisons, might be evoked by a post-transcriptional regulation. This is supported by the number of differentially expressed genes -- especially while comparing CN vs. C limitation -- and the matched protein/mRNA abundance. Nevertheless, to fully confirm this hypothesis further studies should be addressed with more accurate techniques by which thousands of proteins can be quantified and compared [33]. In this study, a surprisingly high number of downregulated genes in CN-limitation relative to C-limitation was found (Additional file 1: Figure S1). This highly differs from the proteome profile (Table 3). In addition, it is interesting to see how *P. putida *KT2442 displays many changes at the transcription level, but at the translation level the final response is quite conservative upon CN limitation. One could propose that under CN-limitation the coupling between anabolism and catabolism is tighter than N-limitation, as it can be seen by the residual biomass formation under the mentioned conditions (Figure 2). Therefore, it seems that dual limitation is more similar to C-limiting than N-limiting cultures, since the proteome profile --which is a closer link than transcriptome to the final phenotype of the cell -- did not have a significant change for the dual limitation relative to strict carbon limitation (Table 3).

#### Metabolomics

Applying metabolome analysis, the peak areas of the Entner-Doudoroff-pathway and TCA cycle intermediates, and amino acids were determined and the mass corresponding masses quantified as described in Materials and Methods. The reproducibility of the analytical protocols (technical reproducibility) was determined for every sample set by establishing the relative standard deviation (RSD) among three samples harvested from the same fermentor. An average RSD of 22% was obtained for the technical reproducibility analyzed by GC-MS. In addition, an average RSD was calculated for specific metabolites in cells grown under the same condition in two independent cultivations (RSD_C-limitation _32%, RSD_CN-limitation _28%, RSD_N-limitation _49%). A Pearson correlation factor of 0.97 between measured metabolites in C- and CN-limitation was determined, which indicates slight differences in metabolite levels in the central carbon metabolism. Between C- and N-limitation, a Pearson correlation factor of 0.84 was obtained, but the biological variances were clearly higher in N-limitation within independent cultivations. Because of the high variability of specific metabolites among biological replicates, most probably due to the sampling procedure since we did not have a fast sampling device for quenching samples, their concentration levels could not be determined. Therefore, these metabolites were excluded from the discussion. Globally, 233 intracellular compounds were found in the chromatogram (containing systemic peaks derivates). According to their retention times and mass spectra, 80 metabolites could be identified by comparison to our in-house libraries.

### Integrated analysis of the various "omics" results under different nutrient limitations

The profile of metabolites in a cell at any given moment is closely related to the perturbation of gene expression and the modulation of protein function [34]. All together, the data from these three levels of cellular response offer a more comprehensive global view of the changes taking place under limitation of carbon and/or nitrogen sources.

#### (i) Nutrient limitation leads to modulation of several transport systems

*Pseudomonas putida *KT2440 possesses approximately 370 cytoplasmic membrane transport systems, a feature that confers a greater transport capability to this versatile bacterium [35]. When comparing N- versus C-limitation, both the ABC transporter that belongs to the ATP-binding cassette superfamily (encoded by PP_4864) and the general amino acid system AapP (encoded by PP_1300) were found, by proteomics, to have increased enzyme levels. In contrast, the genes encoding for proteins responsible for importing specific amino acids, *e.g*. glutamate, aspartate, and other compounds across the membrane, as well as periplasmic ABC transporters (encoded by PP_1071, PP_0282, PP_0112, PP_0885) (Table 3) were clearly downregulated. The dual limitation triggered high induction of only one ABC transporter, LivK (26.4-fold), which is one of the five proteins that form the branched-chain amino acid ABC transporter system and whose transcript level was found also to be induced (Table 2). Another amino acid transporter differentially expressed was LysE (encoded by PP_3021), as well as the urea assimilation system (UreE, UreJ, and UreA). The activation of urease may increase competitive fitness of bacteria under nitrogen-limiting conditions, since urease catalyzes the hydrolysis of urea to yield ammonia and carbamate [36]. For instance, many pathogens are able to colonize several niches in the human body due to the capacity of synthesizing nitrogen from urea, which helps them to overcome nitrogen limitation and thereby increase their potential to thrive in the host [37].

The activation of the major porin protein OprF (encoded by PP_2089) in *Pseudomonas*, was indicated by the variation of the protein production by 2.6-fold and 5.4-fold in CN vs. C and N vs. C, respectively. OprF is involved in conferring several degrees of permeability to the cell [38] and has been described as one the intrinsic factors in *P. aeruginosa *that strongly increases its resistance to antibiotics [39,40]. Another member of the porin family OprD (encoded by PP_1206) was exclusively induced by the dual limitation. The specific porin OprD has been shown to be implicated in the uptake of basic amino acids [41,42], facilitating their diffusion across the membrane. Such behavior indicates that the outer membrane composition follows a sensitive response to different signals, *e.g*. nutrient and/or metabolite concentrations, which is reflected in the modulation of specific transporters as needed. Furthermore, based on the high incorporation level of OprH exclusively under N vs. C limitation, it seems that OprH, an outer membrane protein mainly responsible for membrane stabilization [43,44], has an important role in KT2442 to cope with such elevated concentrations of decanoate used to reach the strict nitrogen limitation.

#### (ii) Fatty acid metabolism and PHA synthesis

Fluorescent pseudomonads are able to metabolize a broad range of fatty acids. The cells first convert the fatty acid to the corresponding acyl-CoA thioester, which is further oxidized by the *β*-oxidation cycle [45]. In this regard, three sets of FadAB genes have been described in KT2440 [46]: FadB and FadA (PP_2136 and PP_2137), FadBx and FadAx (PP_2214 and PP_2215), and PP_2047 and PP_2048, which encode 3-hydroxyacyl-CoA dehydrogenase (FadB-like) and acyl-CoA dehydrogenase (FadE-like), respectively [18,19]. Mutations of those genes showed that FadB and FadA played important roles in fatty acid degradation. However, the deletion of these genes could not completely block the β-oxidation pathway and mutant strains produced PHA with longer chain monomers, likely due to a slower or defective β-oxidation pathway [18,19,47]. Several intermediates from the cycle, such as 2-*trans*-enoyl CoA, (*S*)-3-hydroxyacyl-CoA, and 3-ketoacyl-CoA, may serve as precursors for PHA synthesis. The latter intermediate is also cleaved by a β-ketothiolase to yield acetyl-CoA and acyl-CoA, which have two less carbons compared with the one that initially entered the cycle. It is still unclear which intermediate contributes the most to produce the PHA polymerase substrate (*R*)-3-OH-acyl-CoA.

**Figure 3:**
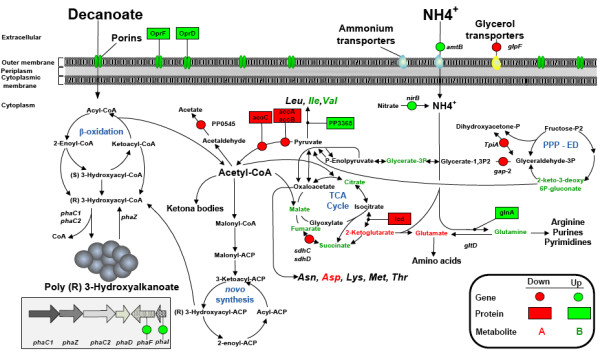
**The scheme shows the changes on the metabolic response at gene, protein, and metabolite level, while growing the cells under carbon-nitrogen in comparison to carbon limitation using decanoate as carbon source**.

Relative to carbon limitation, dual limitation showed no upregulation of enzymes or genes belonging to the β-oxidation pathway (Figure 3). It was not the case for the PHA gene cluster, where the phasins PhaI and PhaF were found upregulated (Table 4). The application of non-standard accumulating conditions e.g. all nutrients in excess or carbon limitation, has been reported to lead to the biosynthesis of polyesters [48,49]. Nevertheless, a clear explanation of this phenomenon remains unknown. Based on our findings, we argue that *P. putida *KT2442 has a metabolic system in which the constant channeling of precursors for PHA accumulation occurs even under carbon limitation since the activity of the β-oxidation pathway (fatty acid metabolism) and the PHA synthases (PhaC) did not change significantly between dual vs. carbon limitation (Table 2, 4, and 3). Dual limitation resulted in 60% of the CDW as PHA. In this case the phasins may segregate and distribute more PHA [50] than under carbon limitation, thus explaining their induction. In contrast, one set of proteins that showed a high expression level under N vs. C were related to the β-oxidation cycle (Table 3). Indeed, this is the main route to metabolize aliphatic fatty acids, where the synthesis of two key metabolites, (*S*)-3-hydroxy-acyl-CoA and acetyl-CoA, occurs simultaneously [45]. The most over-expressed proteins in the fatty acid group, 3-hydroxyacyl-CoA dehydrogenase (encoded by PP_2047) and acetoacetyl-CoA thiolase (encoded by PP_2051), matched the remarkable induction of the gene cluster (PP_2047-PP_2051) at the mRNA level (Table 3). Recent evidence indicates that the protein product of the PP_2047 and PP_2048 open reading frames play very important roles in defining the PHA composition when fatty acids are the carbon source (Liu *et al*., 2011). In this respect, the observed enrichment in C10 found in the chemostat subjected to strict nitrogen limitation could be assigned to this dehydrogenase (PP_2047), since a dramatic shift was obtained in the monomer composition when the gene encoding this enzyme was knocked-out in KT2442 (Liu *et al*., 2011). Nevertheless, under dual limitation a similar monomer composition was found where no overexpression of PP_2047 could be observed at the proteome level. Based on these results, the defined monomer composition is a much more complex mechanism, where nutrients availability seems to be another important factor in controlling the fate of carbon in PHA synthesis. The protein FadA, which is the product of gene PP_2137 (Table 3) that belongs to the first set of genes coding for the *β*-oxidation pathway in *P. putida *converting 3-ketoacyl-CoA into acetyl-CoA and acyl-CoA, is responsible for the last step in the *β*-oxidation pathway and its expression showed a 3.0-fold increase. Furthermore, FadB was identified in two different spots where the average of its protein abundance was about 3-fold. AccC-1 (encoded by PP_0558), an important protein involved in PHA synthesis (Huijberts *et al*., 1992), presented an 8.3-fold induction at N vs. C. It is noteworthy that this enzyme leads the initial step from acetyl-CoA to fatty acid *de novo *synthesis via malonyl-CoA and it is normally activated while growing on non-PHA-related substrates such as sugar and gluconate [51]. Taking all together, we could capture the difference at single molecular levels within the *β*-oxidation pathway which is known to generate intermediates for both biomass and PHA synthesis while fatty acids are supplied as carbon sources [25]. Huijbert and co-workers hypothesized that, at certain level of C/N ratio, the catabolic cycle for fatty acids switches its precursor preference, where instead of replenishing its intermediates for further oxidation, they are converted to PHA monomers. The finding in this study, where the residual biomass is not constant for C/N- and N-limited cultures -- which was not observed in previous reports using *P. putida *GPo1 -- can be explain by the fact that several enzymes (FadB, FadA) and the gene cluster PP_2047-PP_2051 were differentially expressed when comparing N- vs. C-limiting growth. To further investigate which genes contributed most to this difference, we performed a transcriptome analysis comparing N vs. CN limitation using a very strict cut-off (Fold change > 3.5 and a *P *value < 0.03). Only 9 genes fell into this group (Additonal file 3: Table S2). The gene cluster PP_2047-PP_2051 exhibits more than 10-fold change in expression, and especially 3-hydroxyacyl-CoA dehydrogenase (encoded by PP_2047) was induced by 25-folds (Additonal file 3: Table S2). Based on this result, we can postulate that these ORFs are responsible for the diminishing on biomass formation, where the generated precursors within the oxidation cycle are redirected to PHA synthesis. On top of this, AtoB (encoded by PP_2051) is an enzyme that converts acetyl-CoA into acetoacetyl-CoA, a competing pathway for the TCA cycle. By a chain elongation process the acetyl-CoA is further condensed to 3-hydroxyacyl-CoA which is finally involved in PHA synthesis [52], thus explaining the variation on biomass formation while comparing N vs. CN-limited growth in *P. putida *KT2442. With regard to the degradation system of the PHA granule in *P. putida *strains, recent studies have elucidated its influence on the metabolic balance of the cell. A negative PhaZ mutant strain from *P. putida *KT2442 had shown no increase in PHA yield but a reduction in the biomass concentration relative to the wild-type in batch cultures [10]. In the same study, it was demonstrated that PHA metabolism is an ongoing cycle where synthesis and degradation of the polyester is a simultaneous process. The same result was found using *P. putida *U, where the activities of PhaC and PhaZ are concomitantly active [53]. It is worth to mention that in batch processes the kinetic limitation of a single nutrient takes place. As the present study was performed using chemostat cultures, the cells are always exponentially growing, and therefore the physiological state of the cells differs from that shown in batch culture. The expression level of the *phaZ *depolymerase gene was not affected by the dual limitation in comparison to carbon limitation (Table 4). This was not the case when nitrogen was the single limiting nutrient, showing a increase in *phaZ *expression (Table 4). In agreement with one of our previous reports, the transcription levels of *phaC *and *phaZ *are highly correlated [20], thus indicating that such regulatory system is independent of the cultivation mode. This result supports the postulate that at high production rate of PHAs, the expression of the PhaZ is needed for the efficient production of the polyester [54,55].

**Table 4:** Change in protein expression in response to nitrogen and carbon-nitrogen limitation in comparison with carbon-limitation

	Locus tag	Gene symbol		Protein		Transcript	
					
				D.E	**Peptides No.**^**a**^	Fold change	*P*-value
*Differential protein expression of CN vs. C*							
*Energy metabolism*							
ATP synthase F1, α-sub	PP5415	AtpA		*only in CN*	12	ND	ND
Glycogen debranching	PP4055	GlgX		4.5	20	1.9	0.05
Acetolactate synthase	PP3365			3.5	15	-1.0	n.s.
Isocitrate dehydrogenase	PP4011	ICD		-5.0	28	-1.4	n.s.
**Acetoin dehydrogenase^¥ ^α-sub**	**PP0555**	**AcoA**	-4.0		7	**-8.3**	**0**
**Acetoin dehydrogenase β-sub**	**PP0554**	**AcoB**		-5.6	23	**-4.2**	**0**
**Acetoin dehydrogenase**	**PP0553**	**AcoC**		-3.7	10	**-4.0**	**0**
**Nitrogen metabolism**							
**Nitrogen regulatory protein P-II**	**PP5234**	**GlnK**		***only in CN***	**6**	**10.4**	**0**
**Protein synthesis**							
Elongation factor Tu	PP0452	TUF-2		*only in CN*	16	-1.6	n.s.
*Amino acid transport and biosynthesis*							
**Branched-chain amino acid ABC trans**	**PP4841**	**LivK**		**26.4**	**22**	**4.5**	**0**
Glutamine synthetase	PP5046	GlnA		2.2	28	1.0	n.s.
*Signal transduction*							
Response regulator	PP5048	NtrC		6.7	43	1.7	n.s.
*Transport and binding protein*							
Outer membrane porin	PP1206	OprD		2.7	11	-1.1	n.s.
Outer membrane porin	PP2089	OprF		2.6	21	1.2	n.s.
*Purine ribonucleotide biosynthesis*							
adenylosuccinate synthetase	PP4889	PurA		-4.4	16	-1.1	n.s.
**Hypothetical protein**	PP2384			-2.8	6	1.5	n.s.
**Protein fate**	PP0980	PepA		-2.3	30	1.0	n.s.
Differential protein expression of N vs. C							
*Energy metabolism*							
Succinyl-CoA synthetase	PP4185	SucD		*only in N*	8	1.2	n.s.
ATP synthase F1, alpha sub	PP5415	AtpA		*only in N*	13	ND	ND
Glycogen debranching	PP4055	GlgX		12.3	31	2.4	n.s.
Electron transfer flavoprotein	PP4203			4.5	36	0.90	n.s.
Succinate dehydrogenase	PP4191	SdhA		3.9	23	2.8	n.s.
NADH dehydrogenase I	PP4123	NuoF		2.9	15	2.4	n.s.
Glycosyl hydrolase	PP4053			2.6	21	3.0	n.s.
Phosphoenolpyruvate synthase	PP2082	PpsA		2.4	24	1.5	n.s.
**Acetoin dehydrogenase β-sub**	**PP0554**	**AcoB**		***only in C***	**23**	**-4.0**	**0**
**Acetoin dehydrogenase**	**PP0553**	**AcoC**		***only in C***	**10**	**-5.3**	**0**
Malate synthase	PP0356	GlcB		-9.1	9	-1.2	n.s.
Aldehyde dehydrogenase	PP0545	GlcB		-5.9	19	-1.9	n.s.
thioredoxin reductase	PP0786	TrxB		-5.6	11	1.3	n.s.
Isocitrate lyase	PP4116	AceA		-4.2	19	1.3	n.s.
Isocitrate dehydrogenase	PP4011	ICD		-2.1	14	1.1	n.s.
*Fatty acid metabolism*							
**3-hydroxyacyl-CoA dehydrogenase**	**PP2047**			**only in N**	**18**	**20.9**	**0**
**Acetyl-CoA acetyltransferase**	**PP2051**	**AtoB**		**only in N**	**20**	**27.9**	**0**
acetyl-CoA carboxylase	PP0558	AccC-1		8.3	27	1.3	n.s.
Fatty oxidation complex α-sub	PP2136	FadB		3.1	13	2.3	n.s.
3-oxoacyl-CoA thiolase	PP2137	FadA		3.0	23	2.4	n.s.
Fatty-acid CoA ligase	PP0763			-2.9	15	-1.1	n.s.
*Transport and binding protein*							
Outer membrane porin	PP2089	OprF		5.4	17	1.4	n.s.
ABC transporter	PP1726			-25.0	15	1.2	n.s.
*Amino acid transport and biosynthesis*							
Branched-chain amino acid ABC	PP4864			*only in N*	9	-1.1	n.s.
Amino acid biosynthesis	PP5046	GlnA		16.0	17	1.5	n.s.
Hydantoin racemase	PP4310			8.8	15	1.2	n.s.
General amino acid ABC transporter	PP1300	AapP		4.0	16	1.1	n.s.
amino acid ABC transporter	PP1071			*only in C*	23	-2.0	n.s.
amino acid ABC transporter	PP0282			-33.3	15	-1.2	n.s.
Branched-chain amino acid ABC	PP4867			-16.7	11	1.8	n.s.
Dipeptide ABC transporter	PP0885			-5.0	22	1.2	n.s.
Amino acid biosynthesis	PP0671	GlyA-2		-3.6	20	-1.3	n.s.
*Cell envelope*							
**Outer membrane protein H1**	**PP1185**	**OprH**		***only in N***	**8**	**6.6**	**0.01**
**Cellular processes**							
Small multidrug resistance	PP4930			4.9	216	1.1	n.s.
superoxide dismutase	PP0915	SodB		-20.0	4	-1.3	n.s.
*DNA interactions*							
Transcriptional regulator, LysR	PP5375			-12.5	5	-1.5	n.s.
C4-type zinc finger protein	PP4693			-11.1	5	1.4	n.s.
*Protein fate*							
ATP-dependent Clp protease	PP2300			*only in N*	7	1.2	n.s.
*Protein synthesis*							
Elongation factor-G	PP0451	FusA-1		20.0	26	1	n.s.
**Hypotetical protein**	**PP2050**			**9.4**	**10**	**28.4**	**0**
Purine ribonucleotide biosynthesis	PP0722	PrsA		6.6	24	2.0	n.s.
Cell division	PP1342	FtsZ		-7.1	5	1.0	n.s.
Nucleoside interconversions	PP0849	NDK		-3.3	6	1.2	n.s.
Biosynthesis of co-factors	PP0842	IscS-1		-2.9	18	1.3	n.s.
Chemotaxis and motility	PP4366	FliL		-2.3	7	1.3	n.s.
							

^¥ ^Bold font indicates matching between protein and transcript changes

^a^Amount of peptides utilized for protein identification

*ND*, Not determined

*n.s*., *P*-value not significant (> 0.05)

#### (iii) Energy metabolism

##### TCA Cycle

Once the fatty acid is oxidized in subsequent steps within the β-oxidization cycle, acetyl-CoA is produced in the TCA cycle for energy generation and to sustain biomass and maintenance of the cell. Little is known about the regulation of this pathway when fatty acids are used as a carbon source and several nutrient limitations are imposed on the system. This is even more complex since intermediates and co-factors of the Krebs cycle inhibit various enzymatic reactions within the cycle itself. For instance, NADH inhibits the isocitrate dehydrogenase, 2-ketoglutarate dehydrogenase, and isocitrate synthase. A crucial question is how the cells orchestres its metabolism to generate the energy for sustaining growth while simultaneously large quantities of PHA is being synthesized. The F0F1 synthase subunit alpha AtpA (encoded by PP_5415) (oxidative phosphorylation) was overproduced in dual and strict nitrogen limitation as compared to the carbon limitation. According to this finding, oxygen may also have an effect on the PHA production capacity in *P. putida *KT2442, but this statement still needs to be tested. The increased expression of the F0F1-type ATP synthase might be necessary for the cell in order to produce ATP molecules via the loss of proton-motive force, since high PHA yields decrease intracellular levels of ATP [56]. This effect might trigger different expression patterns from enzymes in the TCA cycle which is an important source of ATP generation along with the electron transport chain. As for the activity of the Krebs cycle, several genes were repressed under the dual limitation condition (Table 2). At the protein level only one enzyme, isocitrate dehydrogenase ICD (encoded by PP_4011), showed a fold change less than 2 (Table 3). The deficiency in the TCA cycle was confirmed by the metabolome analysis, where the increase in the pool size of succinate is a direct consequence of the repression of the *sdhC *and *sdhD *genes (Figure 3, Table 2). The quantitative increase of fumarate and malate was also triggered by the dual limitation. As catabolism and anabolism are tightly coupled under carbon limitation, the cell may coordinate the energy requirement in such a way that no overflow of carbon is present. When both carbon and nitrogen are the limiting nutrients simultaneously, the gluconeogenesis pathway responded by repressing the genes *tpiA *and *gap*-2, which coordinate carbon re-funneling in the Krebs cycle and the conversion rates of dihydroxyacetone phosphate and glyceraldehyde3-phosphate (Figure 3). The findings suggest that the carbon catabolic repression observed in the TCA cycle can be attributed to the high expression of the nitrogen regulatory protein NtrC (encoded by PP_5048) (Table 3), which is believed to control such an effect under nitrogen limitation [57].

##### High-energy compounds

**Figure 4:**
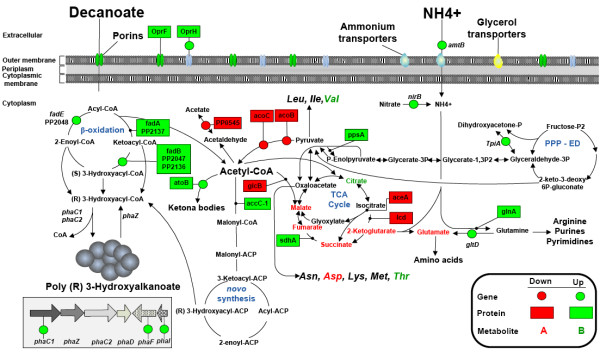
**The scheme shows the changes on the metabolic response at gene, protein, and metabolite level, while growing the cells under strict nitrogen in comparison to carbon limitation using decanoate as carbon source**.

The strict nitrogen-limited cultures differ in their response to energy requirement relative to the CN-limited cultures, principally due to the elevated level of enzymes participating in the Krebs cycle. Succinyl-CoA synthase SucD (encoded by PP_4185), which participates in the synthesis of the high-energy compounds ATP and GTP, was identified in cells only under strict nitrogen limitation. Examining the metabolome, we found a decreased pool size of metabolites down- and up-stream of succinate dehydrogenase, namely succinate and fumarate, respectively (Figure 4). All together it appears that the TCA cycle plays an important role in providing the necessary energy to the cell when PHAs are accumulated in increased proportion.

Employing 2-D gels, we also identified a particular modulation of another important enzyme in the cycle, isocitrate dehydrogenase ICD (encoded by PP_4011), which can be repressed by various mechanisms together with the low expression of ICD, two enzymes of the glyoxylate shunt, isocitrate lyase (encoded by PP_4116) and malate synthase (encoded by PP_0356), were significantly downregulated at strict nitrogen limitation. As such, this phenomenon can be directly related to the repression of the glyoxylate shunt, resulting in the downregulation of malate, which is the main precursor for oxaloacetate synthesis and is known as an essential compound for increasing the flux of acetyl-CoA into the TCA cycle [58]. In this sense, the inactivation of isocitrate lyase and isocitrate dehydrogenase in KT2442 led to the enhancement of PHAs when gluconate was used as the carbon source [58]. The inactivation of the glyoxylate shunt including the downregulation of malate synthase (GlcB) regulates the concentration of malate. Moreover, downregulation of isocitrate dehydrogenase (ICD) had a direct influence on the pool size of 2-ketoglutarate and consequently on glutamate formation. The cell triggered the activation of glutamine synthase (GlnA) to form glutamine to counteract the decreased pool of precursor for the synthesis of amino acids. In this respect, the metabolome profile showed an increase in the concentration of glutamine under CN vs. C limitation. No change in glutamine concentration was observed at N vs. C limitation, although GlnA was also found upregulated. Another metabolite that is directly regulated by the expression of isocitrate dehydrogenase is citrate. Studies on bacteria and yeast subjected to nitrogen limitation [59,60], have shown repression of isocitrate dehydrogenase. Morgunov and co-workers found an increased concentration of citrate in yeast probably caused by the high ratio of NADH/NAD^+^, which inhibits isocitrate dehydrogenase. Notably, after comparing N vs. C and CN vs. C, the pool size of citrate increased under both conditions (Table 5). Although the expression of isocitrate dehydrogenase was found to be significantly reduced under dual limitation, the inactivation was not accompanied by a downregulation of enzymes belonging to the glyoxylate pathway, suggesting a distinct regulatory system in this particular environment.

**Table 5:** Metabolites of known level changes in comparison to carbon-limiting condition

	CN-limitation	N-limitation
**Entner-Doudoroff-pathway**		
Glucose	*up*	*up*
2-Keto-3-deoxy-6Pgluconate	*up*	*n.d*.
3-Phosphoglycerate	*up*	*n.d*.
**TCA cycle**		
Citrate	*up*	*up*
2-Ketoglutarate	*down*	*down*
Succinate	*up*	*down*
Fumarate	*up*	*down*
Malate	*up*	*down*
**Amino acids**		
Aspartate	*down*	*down*
Cysteine	*up*	*n.d*.
Glutamate	*down*	*down*
Glutamine	*up*	*up*
Glycine	*up*	*up*
Isoleucine	*up*	*n.d*.
Ornithine	*n.d*.	*down*
Serine	*n.d*.	*up*
Threonine	*n.d*.	*up*
Tyrosine	*up*	*down*
Valine	*up*	*up*

*n.d*., Not defined

##### Electron transport chain

Another important finding while comparing N vs. C limitation was the high induction of genes encoding the cytochrome o ubiquinol oxidase Cyo (encoded by PP_0813-0814) (Table 2). Cyo is a terminal oxidase of the electron transport chain which is believed to sense the energetic state of the cell, thus controlling the catabolic pathways and the expression of different genes [61]. In addition, terminal oxidases are highly regulated by oxygen concentration [62,63]. Due to the high oxygen concentration supplied to the bioreactor in order to keep the oxygen tension above 20%, it is most likely that such an environment affects the composition of the electron transport chain. One possible explanation for the reorganization of the terminal oxidase under strict nitrogen limitation, is to optimize the energy generation in the cell [63] since a remarkable energy accumulation in the polyester form is present. In addition, Cyo levels have been shown as being responsible for decreasing the expression level of many promoters *in P. putida *including the PalkB promoter (for alkane assimilation). Furthermore, rather than a carbon catabolic repression, this effect has been postulated as a general physiological control mechanism [64]. Whether or not Cyo modulates the expression of genes under strict nitrogen limitation is still a question to be answered. There is a clear correlation between the TCA cycle and the electron transport chain, as shown by the high expression of the protein succinate dehydrogenase (4-fold change) which stimulates the cytochrome o ubiquinol oxidase by supplying electrons directly to it.

The overflow metabolism is one of the repeated trends of chemostats subjected to strict nitrogen limitation [14]. Since the exo-metabolite profile did not contain any secondary metabolites under this condition, *P. putida *KT2442 seems to arrange its metabolism in such a manner that it blocks energy-spilling by strongly repressing enzymes involved in the pyruvate and acetate pathways (*acoA, acoB, acoC*, PP_0545) (Table 3), thus preventing the shortage of partially oxidized metabolites [65]. This may be desirable because of the huge channeling of the consumed carbon (reflected in energy storage) towards polyester synthesis. Despite the dowregulation of those enzymes in the pyruvate and acetate pathways where acetyl-CoA is needed, there was a high expression of isoacetyl-CoA carboxylase and biosynthetic thiolase, which are the first enzymes responsible for channeling acetyl-CoA to *de novo *fatty acid and ketona bodies biosynthesis, respectively. Interestingly, it seems most likely that *P. putida *KT2442 channels the remaining acetyl-CoA into storage compounds by activating those pathways to save energy for later consumption (Figure 4).

#### (iv) Nitrogen and amino acid metabolism

As seen in Tables 2 and 3, the nitrogen regulatory protein P-II GnlK (encoded by PP_5234) showed a large change in both mRNA abundance and protein level, when *Pseudomonas putida *was subjected to dual limitation. This was not the case when we examined the proteome profile from strict nitrogen-deprived cells, since the spot for this enzyme did not show differential expression compared to the carbon-limited cultures. The expression of GnlK is led by the nitrogen availability via the nitrogen transcriptional regulator NtrC in *P. putida *KT2442 [66]. We also found that KT2442 displays the upregulation of NtrC when both carbon and nitrogen were completely depleted (Table 3). NtrC also modulates the expression of the *glnA *gene (Hua *et al*., 2004), which is positioned upstream in the *glnA-ntrAB *operon in *Escherichia coli*. Hervas and co-workers showed NtrC-dependency for the (*glnA*) gene in KT2442, postulating that it could be part of an operon together with (*ntrB*) and (*ntrC*) genes, like in enterobacteria. Notably, GlnA exhibited the highest levels of induction among the proteins associated to amino acid biosynthesis (Table 3) in the case of strict nitrogen limitation. The condition also caused significant repression of several enzymes that were identified as serine hydroxymethyltransferase GlyA, putative hydantoin racemase (encoded by PP_4310), and branched-chain amino acid aminotransferase Ilv*E*. In the valine, leucine, and isoleucine metabolic pathway IlvE has a double function to either act together with leucine dehydrogenase in the synthesis/degradation of leucine or in the degradation of valine and isoleucine to glutamate by transferring nitrogenous groups. Another enzyme responsible for the turnover of certain amino acids in the same family is GlyA, which carries out the conversion of glycine by transferring one-carbon group, giving serine as a product. Furthermore, it catalyzes the reaction of glycine with acetaldehyde to form threonine. Particularly, the downregulation of the periplasmic glutaminase-asparaginase protein occurred solely under strict nitrogen limitation. This protein is responsible for the uptake of the acidic amino acids, e.g. aspartate and glutamate [67]. Carbon catabolite repression of the periplasmic glutamate/aspartate transporter took place when KT2440 was exposed to glucose or mono- and dicarboxylic acids [68]. However, the metabolome profile showed a decrease in the pool size of fumarate and succinate (Table 5). Therefore, there may be another system which drives the transport selectivity described here.

#### (v) Stress response proteins

Besides their primary feature as a reservoir of energy and carbon, PHAs have displayed a key role in conferring robustness to the bacteria under unfavorable conditions, such as temperature, UV irradiation, chemicals, and osmotic pressure [69]. In contrast, based on the high polyester accumulation within the cell (Table 1), PHAs have also been postulated to act as a stressor, especially concerning protein synthesis and the activation of various protective proteins[70]. It is worth noting that all studies which stated such a stress produced by the polyester inclusion to the cell, have been performed in metabolically engineered *E. coli *[70-72].

In the current study a stress response of the cell while growing under CN- and N-limitation can be seen. The high expression level of elongation factor enzymes is the first indicator of such an effect. The protein abundance of TuF-2 (encoded by PP_0452, Table 3), one of the Tu- elongation factor enzymes, was extremely high under CN limitation. Besides its primarily role of binding and transporting the proper codon-specified aminoacyl-tRNA to the aminoacyl site of the ribosome, TuF-2 also has chaperon-like functions that enhance protein folding and stress protection[73]. Strict nitrogen-limited cultures exhibit overexpression of the elongation factor FusA-1 (encoded by PP_0451, Table 3), which is a GTPase (classified as a G-elongation factor) that is involved in the translocation of bacterial ribosomes along messenger RNA during protein biosynthesis [74]. On the other hand, the protease enzyme PP_2300, which is involved in hydrolysis of proteins to small peptides, was strongly induced in the presence of excess carbon (Table 3). So far, the decreased expression level of Tu-elongation factor has been related to the reduced capability of a metabolically engineered *Escherichia coli *to synthesize proteins (Han *et al*., 2001). However, *E. coli *does not naturally accumulate PHAs, therefore it is not surprising that KT2442 showed a different proteome pattern for these elongation factor enzymes. Indeed, the normal protein synthesis machinery is affected by the shortage of nitrogen; thereby the cell may react in such a way that guarantees the biosynthesis of the main building blocks (amino acids) of these macromolecules. In addition, the induction of the gene encoding the chaperonin 10 GroES (Table 2) was another metabolic adaptation against the stress produced by the nitrogen limitation. Han and colleagues also observed a high level expression of several heat shock proteins, arguing that the polyester inclusion disturbs the normal intracellular architecture due to its direct contact with the chromosome, resulting in activation of the heat shock response. Nevertheless, GroES also mediates protein folding. We suggest that this may be the principal reason for its incorporation since the genes coding the phasin enzymes were found to be induced (Table 1). The phasins prevent the disturbance of intracellular architecture by forming a protein layer covering the hydrophobic surface of the PHAs, thus creating a protective barrier between the core of the granule and the cytoplasm [6]. Very recently, we have demonstrated that the phasin PhaF is involved in granule cell localization and granule segregation during cell division by interaction with the chromosome [50]. Based on our results, we cannot elucidate whether or not phasins enzymes, PhaF or PhaI, have an influence on stress protein formation. To address this question, mutants strain deficient of genes encoding for PhaF and PhaI should be generated and assessed, by comparing them against the wild-type strain at the proteomic level under well-defined conditions (chemostat).

## Conclusions

By combining measurements of transcriptomics, proteomics, and metabolomics, under well-controlled nutrient limitations, we have shown that the underlying cellular wiring is remarkably different when two nutrients are limiting at the same time, as compared to those under single limitation. We have captured the response of *P. putida *KT2442 to the shift in resource distribution and how these changes activate/repress several metabolic pathways along with transporter systems. We have found that the modulation of the expression of outer membrane proteins -- in particular porins -- under nitrogen and dual limitation correlates with the specific uptake rate of decanoate within the chemostat. We have shown that these two conditions also promote the overexpression of many transporters for scavenging nitrogen sources. The PHA content under nitrogen limitation was found to be the highest, followed by dual limitation and carbon limitation. The residual biomass was not constant for dual- and strict nitrogen-limiting growth, showing a different feature in comparison to other *P. putida *strains. Furthermore, the high accumulation of PHA under carbon limitation is a result of the constant channeling of precursors for PHA biosynthesis since no significant difference could be observed within the *β*-oxidation pathway at any omic level, when compared to dual limitation. Regarding energy generation, genes and proteins belonging to the electron transport chain system were found significantly upregulated when there was a considerable amount of PHA stored in the cell. To overcome the stress caused by the shortening of precursors for amino acid synthesis (nitrogen) and, consequently protein production, elongation factors along with chaperons guarantee the biosynthesis of the main building blocks (amino acids) of these macromolecules.

The systems approach applied in this study has enabled us to gain substantial insights into the metabolic adaptation of *Pseudomonas putida *KT2442 to nutrient limitation. Owing to its comprehensiveness and integration, the knowledge generated will be of great assistance on the development of further metabolic engineering work in this versatile organism to both enhance and diversify the production of PHAs. Our findings, especially concerning the dual limitation that uses both the carbon and nitrogen sources in the most economical manner, have brought an alternative to fulfill one of the remaining gaps in further improving industrial production of PHAs.

## Methods

### Culture conditions and bacterial strain

*Pseudomonas putida *KT2442 [75] was grown in a defined mineral medium (MM) consisting of (per liter) 12.8 g Na_2_HPO_4_.7H_2_O, 3 g KH_2_O_4_, 1 g NH_4_Cl, 0.5 g NaCl, supplemented with 0.12 g of MgSO_4_.H_2_O, and trace elements (mg·l^-1^): 6.0 FeSO_4_.7H_2_O, 2.7 CaCO_3_, 2.0 ZnSO_4_.H2O, 1.16 MnSO_4_.H_2_O, 0.37 CoSO_4_.7H_2_O, 0.33 CuSO_4_.5H_2_O, 0.08 H_3_BO_3 _and 0.1% (v/v) TEGO antifoam D 2310 (EVONIK Industries, Essen, Germany). Sodium decanoate (98% purity, Sigma-Aldrich) was used as a single carbon source in concentrations ranging from 1.90 to 8.70 g·l^-1 ^(Table 1). For the preparation of the feeding solution, MM was mixed with sodium decanoate and TEGO antifoam. After mixing it, the solution was autoclaved for 25 min at 121°C. Once the bottle reached room temperature, it was placed into the clean bench where sterile trace elements solution and MgSO_4_.H_2_O (by filtration) were added. Continuous cultivations were carried out under aerobic conditions at a dilution rate (*D*) of 0.1 h^-1^, working volume of 0.8 liter in 1 liter top-bench BIOSTAT B1 bioreactor (Sartorius B Systems GmbH, Melsungen, Germany) at 30°C, constant stirring speed 700 r.p.m., and the pH was maintained at 7.0 by adding 2 M H_2_SO_4_. The working volume was kept constant by removing the fermentation broth through a peristaltic pump and recording the weight with a balance placed under the bioreactor. The aeration rate was set at 1 l·min^-1 ^using a mass flow controller (PR4000, MKS Instruments, Wilmington, MA, USA), a mixture of air and pure oxygen was used to ensure that the dissolved oxygen was above 30% of air saturation. A paramagnetic gas analyzer (Servomex Xentra 4100, USA) to record online the concentrations of carbon dioxide and oxygen in the course of the process was coupled to the gas outlet of the bioreactor.

### Analytical procedures

Once the considered steady state was attained, - *i.e*. that the optical density of the biomass, dissolved oxygen concentration, and exhaust gas concentration remained constant for at least four residence times - samples from chemostat cultures were taken using a peristaltic pump and processed as follows:

#### Cell and ammonium concentration

Cellular dry weight was determined by centrifuging 5 ml samples of the culture broth for 10 min at 4°C and 10,000 rpm (centrifuge 5810 R, Eppendorf, Germany) in pre-weighed tubes, washing the cell pellets once with distilled water and drying them at 80°C until a constant weight was obtained. Cell growth was recorded by measuring the optical density at 600_nm _(Ultraspect 2000 UV/VIS, Hitachi, Japan). The ammonium concentration in the cell-free supernatant was measured using the LCK 303 kit of Hach Lange (Danaher, USA). The detection limit of this method was 1.9 mg of N/liter.

#### Fatty acid and PHA analysis

PHA compositions of the polymer produced, as well as the cellular PHA content and fatty acid concentration were determined by gas chromatography (GC) and mass spectrometry (MS) of the methanolyzed fatty acid and polyester. Methanolysis procedure was carried out by suspending 5-10 mg of lyophilized aliquots in 2 ml of chloroform and 2 ml of methanol containing 15% sulfuric acid and 0.5 mg ml^-1 ^3-methylbenzoic acid (internal standard) and then incubated at 100°C for 4 h. After cooling, 1 ml of demineralized water was added and the organic phase containing the resulting methyl esters of monomers was analysed by GC-MS [76]. An Agilent (Waldbronn, Germany) series 7890A coupled with a 5975 C MS detector (EI, 70 eV) and a split-splitless injector were used for analysis. An aliquot (1 ml) of organic phase was injected into the gas chromatograph at a split ratio of 1:50. Separation of compounds was achieved using a HP-5 MS capillary column (5% phenyl-95% methyl siloxane, 30 m × 0.25 mm i.d. × 0.25 mm film thickness). Helium was used as carrier gas at a flow rate of 0.9 ml min^-1^. The injector and transfer line temperature were set at 275°C and 300°Crespectively. The oven temperature programme was: initial temperature 80°C for 2 min, then from 80°C up 150°C at a rate of 5°C min^-1 ^and finally up 200°C at a rate of 10°C min^-1^. EI mass spectra were recorded in full scan mode (*m/z *40-550).

#### Exometabolite profile

1D ^1^H nuclear magnetic resonance spectra were recorded on a Bruker AVANCE DMX600 NMR spectrometer at 300 K of aqueous centrifuged supernatant containing 10% D_2_O to give a final volume of 0.66 ml. The water signal was suppressed using standard Bruker software. For comparison purposes, spectra of solutions of initial medium containing antifoam and sodium decanoate were recorded. In order to acquire an appropriate signal-to-noise ratio, spectra were recorded under standard conditions (sweep width: 20 ppm, acquisition time: 1.36 s, pulse delay: 1 s, number of scans: 1400, Bruker program noesypr1d).

### Transcriptomics

Aliquots of 10 ml of culture broth were placed in RNAprotect buffer (Qiagen), cell pellets were further frozen at -80°C. Isolation of total RNA was performed using RNeasy kits (Qiagen), according to the instructions provided by the manufacturer. Progenika Biopharma (Vizcaya, Spain) *P. putida *Oligonucleotides Arrays were used for all transcriptional analyses. Fluorescently labelled cDNA for microarray hybridizations was obtained by using the SuperScript Indirect cDNA Labeling System (Invitrogen), as recommended by the supplier. In brief, 20 μg of total RNA was transformed to cDNA with Superscript III reverse transcriptase using random hexamers as primers, and including aminoallyl-modified nucleotides in the reaction mixture. After cDNA purification, the Cy3 or Cy5 fluorescent dyes (Amersham Biosciences) were coupled to the amino-modified first-strand cDNA. Labeling efficiency was assessed using a NanoDrop ND1000 spectrophotometer (NanoDropTechnologies). Equal amounts of Cy3- or Cy5-labelled cDNAs, one of them corresponding to the control and the other one to the condition to be analyzed, were mixed and dried in a Speed-Vac. The scanning was done with a GenePix 400B Scanner (Molecular Devices Corporation, Sunnyvale, California, USA.) at a 10 μm resolution. The images were quantified with GenePix Pro 5.1. Images from Cy3 and Cy5 channels were equilibrated and captured with a GenePix 4000B (Axon) and spots were quantified using GenPix Pro 5.1 software (Axon). The microarrays were analyzed using various packages from the Bioconductor suite [77]. The results of image analysis were read in using the 'limma' package. The quality of the chips was analyzed with the 'arrayQualityMetrics' package [78]. The intensity values were background-corrected using the "normexp" method of the limma package [79] and normalized with the variance stabilization method [80]. The significantly differentially expressed genes were identified by fitting the linear model (using the functions 'lmFit' and 'eBayes' from the 'limma' package) [81]. Genes for which the adjusted p-value (by Benjamini-Hochberg method) was lower than 0.05 and the fold change exceeded 2 in either direction were assumed to be significantly expressed.

### Proteomics

For protein extraction, cells were centrifuged at 8000 *g *for 15 minutes (centrifuge 5810 R, Eppendorf, Germany), the pellet was washed twice with PBS solution (pH 7.4). The resulting pellet was resuspended in lysis buffer consisting of 7 M urea, 2 M thiourea, 4% w/v CHAPS, 20 mM Tris base and 30 mM 1.4 dithiothreitol (DTT), together with protease inhibitor cocktail tablets. The lysis was complemented by sonication (Labsonic U, B. Braun, Germany. The resulting solution was ultracentrifuged (Sorval ultracentrifuge OTD-Combi, Thermo Electron, Germany) at 12000 *g*, 4°C for 30 min. The supernatant, corresponding to the soluble protein fraction, was aliquoted and further precipitated by using the 2-D Clean-Up Kit (GE Healthcare, USA). Analytical determinations were carried out with 100 mg of protein mixture determined by Bradford (Bio-Rad protein assay, Bio-Rad, USA), diluted up to 300 ml with rehydration solution (7 M urea; 5% w/v Serdolit; 2 M thio-urea; 4% w/v CHAPS; 20 mM Trizma base) in the presence of ampholytes and under reducing conditions, on ReadyStrip IPG strips, 17 cm, pH 3-10 (Bio-Rad, USA). Passive rehydration was carried out for 2 h at 20°C on the focusing tray. Samples were covered with silicon oil to avoid dehydration. Active rehydration was performed at 50 V for 12 h. Isoelectric focusing was done at a final voltage of 10 000 V on ProteanIEF cell (Bio-Rad, USA) until reaching 75 kVh. Focused samples were stored at -70°C until the second dimension step. Focused ReadyStrip IPG strips were equilibrated first in equilibration buffer containing 6 M urea, 0.375 M Trizma base (pH 8.6), 30% v/v glycerin, 2% w/v SDS and 2% w/v DTT and later in the same buffer replacing DTT with 2.5% w/v iodoacetamide. After equilibration, second-dimension separation was performed on 12-15% gradient SDS-polyacrylamide 20 × 20 cm gels with the focused sample embedded in 0.5% IEF agarose in a Protean Plus Dodeca Cell (Bio-Rad, USA) at 100 V overnight. The gels were fixed in 10% trichloroacetic acid solution for a minimum of 3 h, stained with 0.1% w/v Coomassie Brilliant Blue G-250 solution overnight and finally de-stained with distilled water. Images of the 2-DE gels were captured with a molecular imager GS-800 calibrated densitometer (Bio-Rad, USA) and processed using Phoretix 2D image analysis software version 2004 (NonLinear Dynamics, UK) for protein DE analysis. Differential expression was defined as the ratio of spot protein expression in a comparative image to the expression of a corresponding spot in a reference image. Up- and down-regulation of protein expression were considered when the *p *value was less than 0.05, and twofold (or higher), and 0.5-fold (or lower) DE values, correspondingly. Protein spots were excised manually from the gels. Spots were de-stained and digested overnight using sequence grade modified trypsin (Promega, USA). The peptides were eluted and desalted with ZipTip (Millipore, USA). For MALDI-TOF analysis, the samples were loaded along with a-cyano-4-hydroxycinnamic acid matrix. The target was then analyzed using an Ultraflex II ToF (Bruker Daltonics, USA). The resulting spectra were used for Peptide Mass Fingerprint (PMF) and analyzed using FlexAnalysis 2.0 and Biotools 2.2 software (Bruker Daltonics). Proteins were identified using an in-house-licensed Mascot search engine (version 2.1.0, Matrix Science, U.K.). The P. putida KT2440 database was searched using the MALDI TOF-MS data with carbamidomethyl cysteine as a fixed modification and oxidized methionine as a variable modification. Trypsin was specified as the proteolytic enzyme and up to two missed cleavages were allowed.

### Metabolomics

For metabolite extraction, cells were centrifuged at 4629 *g*, 4°C for 3 minutes (centrifuge 5810 R, Eppendorf, Germany). The supernatant was discarded and the cell pellet was resuspended in 10 ml pre-cooled 0.9% NaCl (w/v). After further centrifugation the supernatant was discarded to complete the washing step. Furthermore, the resulting cell pellet was resupended in 1.5 ml cold methanol containing 60 μl ribitol (0.2 g·l^-1^) as internal standard. To achieve cell lysis the tubes were incubated in an ultrasonic bath (15 min, 70°C). After the samples were cooled down for 2 min by placing them on ice, 1.5 ml of deionized H_2_O was added and vortexed for 30 s. The addition of 1 ml chloroform was followed by mixing and centrifugation (9000 *g*, 4°C, 5 min); 1 ml of the polar phase was subjected to a centrifugal-vacuum concentrator to allow evaporation. For derivatization, dried pellets were redissolved in 20 μl pyridine, containing 20 mg ml^-1 ^methoxyamine hydrochloride, at 30°C for 90 min under shaking. After adding 32 μl N-methyl-N-trimethylsilyltrifluoroacetamide, samples were incubated at 37°C for 30 min, followed by incubation at room temperature for 90 min under continuous shaking. Finally, the samples were centrifuged at 14 000 *g *for 5 min and the supernatant was used for GC-MS analysis. All samples were analysed within 24 h after derivatization. A retention index marker (n-alkanes ranging from C10...C36 in cyclohexane) was used to convert retention times to retention indices.

GC-MS analysis was performed on a FinniganTrace mass spectrometer (ThermoFinnigan, San Jose, USA). In summary, 1 μl of the derivatized samples was injected in randomized sequence into a programmed temperature vaporizer in split mode (1:25) at 70°C. After an initial time of 0.2 min the injector was ramped at 14°C s^-1 ^to a final temperature of 280°C and held for 5 min. The gas chromatograph was equipped with a J&W DB-5MS column (30 m × 0.25 mm ID, 0.25 μm film thickness). The GC was operated at constant flow of 1 ml min^-1 ^helium. The temperature program started at 70°C, held for 1 min, followed by a temperature ramping of 10°C min^-1 ^to a final temperature of 325°C, which was held constant for 6 min. The transfer line temperature was set to 275°C. Ion source temperature was adjusted to 200°C. Full-scan mass spectra of m/z 40...460 were collected at an acquisition rate of 2.5 scans sec^-1^. Solvent delay time was 4.5 min. For data acquisition Xcalibur 1.2 (Thermo Scientific) was used.

All chromatograms were processed using MetaboliteDetector [82] for targeted analysis. The software supports automatically deconvolution of all mass spectra from a chromatogram and calculates the retention indices. The obtained mass spectra were matched against the reference with a minimum match factor of 0.75. Compounds were annotated by retention index and mass spectra comparison to our in-house library. Selected fragment ions unique for each individual metabolite were used for quantification. Finally, each compound was normalized by peak area from the internal standard (ribitol) and given cell dry weight.

### Calculations

In the chemostat, the physiological parameters, as well as the PHA productivity where calculated according to the following equations:

(1) $$Y_{X/C}=\frac{\Delta X}{\Delta C}\;\left[\text{g}\cdot {\text{g}}^{-\text{1}}\right]$$

(2) $$Y_{X/N}=\frac{\Delta X}{\Delta N}\;\left[\text{g}\cdot {\text{g}}^{-\text{1}}\right]$$

(3) $$q_{C/\hat{x}}=\frac{\Delta C\cdot D}{X_{PHA-free}}\;\left[\text{g}\cdot {\left(\text{g}\cdot \text{h}\right)}^{-\text{1}}\right]$$

(4) $$q_{PHAI\hat{x}}=\frac{PHA\cdot D}{X_{PHA-free}}\;\left[\text{g}\cdot {\left(\text{g}\cdot \text{h}\right)}^{-\text{1}}\right]$$

The nutrient yield coefficient for carbon and nitrogen were used to calculate the boundaries according to the procedure described by Egly and Quayle (1986):

(5) $$\frac{C_{0}}{N_{0}}≅\frac{Y_{X/N}\cdot 14}{Y_{X/C}\cdot 12}\;\left[\text{mol}\cdot \text{mo}{\text{l}}^{-\text{1}}\right]$$

## Competing interests

The authors declare that they have no competing interests.

## Authors' contributions

IPC designed the study, carried out the experiments including fermentations, analyzed the transcriptome and proteome data, interpreted the data and composed the manuscript, IFE carried out the PHA characterization and transcriptome analysis, CJ performed the metabolome analysis, DS provided discussion in the metabolome analysis, JP carried out the computational work involving the statistical analysis, CL critically revised and corrected the manuscript, MAP provided critical discussion and corrected the manuscript, VMDS revised and corrected the manuscript. All authors read and approved the final manuscript.

## Supplementary Material

Additional file 1 **Figure S1 Genes differentially expressed in response to the limitation of nitrogen and carbon-nitrogen limitation**. The generated functional categories groups were based on COG data. Click here for file

Additional file 2 **Table S1 Transcriptomic data of genes differentially expressed with a fold change above 2 and below -2 and a *P *value below 0.05**. Comparison 1: CN- vs. C-limited cultures. Comparison 2: N- vs. C- limited cultures. Click here for file

Additional file 3 **Table S2 Proteomics data of proteins that were differentially expressed**. Click here for file

Additional file 4 **Table S3 Transcriptomic data of genes differentially expressed with a fold change above 3 and a *P *value below 0.02**. Nitrogen- vs. dual-nutrient-limited cultures. Click here for file
