# Supplementary material for: The metabolic response of P. putida KT2442 producing high levels of polyhydroxyalkanoate under single- and multiple-nutrient-limited growth: Highlights from a multi-level omics approach
Source: Microb Cell Fact. 2012 Mar 20;11:34. doi: 10.1186/1475-2859-11-34 (PMC3325844; doi:10.1186/1475-2859-11-34)
Supplement: Additional file 1 — Figure S1 Genes differentially expressed in response to the limitation of nitrogen and carbon-nitrogen limitation. The generated functional categories groups were based on COG data. [file 1475-2859-11-34-S1.DOC]

**Additional Files**

Supplementary Table S1. Transcriptomic data of genes differentially expressed with a fold change above 2 and below -2 and a *P* value below 0.05. Comparison 1: CN- vs. C-limited cultures. Comparison 2: N- vs. C- limited cultures.

Comparison 1

| ***Locus name*** | ***Gene Name*** | ***Log change*** | ***Fold change*** | ***p-value*** |
| --- | --- | --- | --- | --- |
| PP_0555 | acetoin dehydrogenase alpha subunit gb|AE015451.1|:c645014-644037 | -3.12 | **-8.68** | 0.00 |
| PP_4006 | arginine-tRNA-protein transferase-related protein gb|AE015451.1|:4516135-4516842 | -2.84 | **-7.14** | 0.00 |
| PP_0600 | ribosomal protein S20 gb|AE015451.1|:c707346-707068 | -2.75 | **-6.73** | 0.00 |
| PP_0556 | acetoin catabolism protein gb|AE015451.1|:c646092-645037 | -2.18 | **-4.55** | 0.00 |
| PP_0554 | acetoin dehydrogenase beta subunit gb|AE015451.1|:c644003-642981 | -2.04 | **-4.12** | 0.00 |
| PP_0553 | acetoin dehydrogenase dihydrolipoamide acetyltransfe gb|AE015451.1|:c642984-641878 | -2.01 | **-4.03** | 0.00 |
| PP_1037 | phosphoribosylformylglycinamidine synthase gb|AE015451.1|:1184794-1188693 | -2.00 | **-3.99** | 0.01 |
| PP_2297 | integrative genetic element Ppu40 integrase gb|AE015451.1|:2624995-2625819 | -1.99 | **-3.97** | 0.00 |
| PP_2435 | cysteine desulfurase gb|AE015451.1|:c2782362-2781145 | -1.96 | **-3.89** | 0.00 |
| PP_3504 | conserved hypothetical protein gb|AE015451.1|:3974810-3975076 | -1.96 | **-3.88** | 0.01 |
| PP_2149 | glyceraldehyde 3-phosphate dehydrogenase gb|AE015451.1|:2455283-2456746 | -1.89 | **-3.70** | 0.00 |
| PP_4872 | conserved hypothetical protein gb|AE015451.1|:c5540982-5538628 | -1.85 | **-3.59** | 0.00 |
| PP_0557 | acetoin catabolism regulatory protein gb|AE015451.1|:646376-648229 | -1.81 | **-3.50** | 0.00 |
| PP_2423 | conserved hypothetical protein gb|AE015451.1|:c2771139-2770240 | -1.75 | **-3.37** | 0.02 |
| PP_4715 | triosephosphate isomerase gb|AE015451.1|:c5362357-5361602 | -1.72 | **-3.29** | 0.01 |
| PP_4793 | conserved hypothetical protein gb|AE015451.1|:c5456039-5455725 | -1.72 | **-3.28** | 0.00 |
| PP_0389 | ribosomal protein S21 gb|AE015451.1|:c475503-475288 | -1.71 | **-3.28** | 0.00 |
| PP_5214 | transcription termination factor Rho gb|AE015451.1|:c5948464-5947205 | -1.71 | **-3.27** | 0.02 |
| PP_4174 | 3-hydroxydecanoyl-(acyl-carrier-protein) dehydratase gb|AE015451.1|:4716908-4717423 | -1.70 | **-3.26** | 0.01 |
| PP_3954 | periplasmic binding protein putative gb|AE015451.1|:4460534-4461484 | -1.70 | **-3.24** | 0.04 |
| PP_2437 | acyl-CoA dehydrogenase putative gb|AE015451.1|:2783545-2784789 | -1.68 | **-3.20** | 0.00 |
| PP_1016 | sugar ABC transporter permease protein gb|AE015451.1|:1159286-1160194 | -1.67 | **-3.17** | 0.04 |
| PP_0545 | aldehyde dehydrogenase family protein gb|AE015451.1|:c633438-631918 | -1.66 | **-3.17** | 0.01 |
| PP_4737 | D-lactate dehydrogenase putative gb|AE015451.1|:5386489-5389299 | -1.66 | **-3.15** | 0.01 |
| PP_2141 | conserved hypothetical protein gb|AE015451.1|:2444126-2444359 | -1.65 | **-3.14** | 0.00 |
| PP_5224 | conserved hypothetical protein gb|AE015451.1|:c5960294-5960070 | -1.60 | **-3.03** | 0.01 |
| PP_0469 | ribosomal protein L6 gb|AE015451.1|:557869-558402 | -1.59 | **-3.02** | 0.00 |
| PP_5127 | conserved hypothetical protein gb|AE015451.1|:5847820-5849016 | -1.58 | **-2.99** | 0.03 |
| PP_3560 | transcriptional regulator LysR family gb|AE015451.1|:4039141-4040004 | -1.55 | **-2.93** | 0.01 |
| PP_1076 | glycerol uptake facilitator protein gb|AE015451.1|:c1236186-1235335 | -1.54 | **-2.91** | 0.03 |
| PP_1083 | bacterioferritin-associated ferredoxin putative gb|AE015451.1|:c1242882-1242664 | -1.54 | **-2.91** | 0.01 |
| PP_1430 | alginate biosynthesis negative regulator serine prot gb|AE015451.1|:1629584-1631062 | -1.53 | **-2.89** | 0.03 |
| PP_0551 | MORN domain protein gb|AE015451.1|:c640742-638814 | -1.52 | **-2.87** | 0.04 |
| PP_0765 | conserved hypothetical protein gb|AE015451.1|:880484-882370 | -1.46 | **-2.74** | 0.01 |
| PP_0932 | glutamyl-tRNA(Gln) amidotransferase C subunit gb|AE015451.1|:c1075177-1074869 | -1.45 | **-2.74** | 0.01 |
| PP_1099 | cold-shock domain family protein gb|AE015451.1|:c1257128-1256919 | -1.45 | **-2.74** | 0.01 |
| PP_2086 | crfX protein gb|AE015451.1|:2378443-2378715 | -1.42 | **-2.68** | 0.02 |
| PP_0974 | hypothetical protein gb|AE015451.1|:1113202-1113531 | -1.42 | **-2.68** | 0.01 |
| PP_2088 | RNA polymerase sigma factor SigX gb|AE015451.1|:2379632-2380222 | -1.42 | **-2.68** | 0.01 |
| PP_4517 | conserved hypothetical protein gb|AE015451.1|:5131645-5132232 | -1.41 | **-2.65** | 0.02 |
| PP_3071 | acetoacetyl-CoA synthetase putative gb|AE015451.1|:c3455887-3453935 | -1.41 | **-2.65** | 0.01 |
| PP_1766 | initiation factor 2 subunit family gb|AE015451.1|:1969627-1970703 | -1.40 | **-2.65** | 0.01 |
| PP_2258 | sensory box protein gb|AE015451.1|:2574821-2576509 | -1.40 | **-2.64** | 0.04 |
| PP_4147 | peptide ABC transporter periplamic peptide-binding p gb|AE015451.1|:4685186-4687021 | -1.40 | **-2.64** | 0.01 |
| PP_2853 | conserved hypothetical protein gb|AE015451.1|:c3257150-3255870 | -1.39 | **-2.63** | 0.02 |
| PP_1799 | GDP-mannose 4 6 dehydratase gb|AE015451.1|:2021899-2022966 | -1.39 | **-2.63** | 0.01 |
| PP_4439 | ISPpu14 transposase Orf3 gb|AE015451.1|:5033959-5035494 | -1.39 | **-2.62** | 0.02 |
| PP_3402 | hypothetical protein gb|AE015451.1|:c3854191-3853796 | -1.37 | **-2.59** | 0.01 |
| PP_2874 | hypothetical protein gb|AE015451.1|:3275984-3276295 | -1.37 | **-2.59** | 0.01 |
| PP_3557 | methyl-accepting chemotaxis transducer gb|AE015451.1|:4034947-4037091 | -1.36 | **-2.56** | 0.04 |
| PP_5067 | potassium efflux system protein KefA putative gb|AE015451.1|:5781517-5784825 | -1.35 | **-2.56** | 0.04 |
| PP_5124 | ferredoxin 4Fe-4S gb|AE015451.1|:5845668-5845919 | -1.35 | **-2.55** | 0.04 |
| PP_3319 | sensory box protein/GGDEF domain protein gb|AE015451.1|:3754557-3756758 | -1.35 | **-2.55** | 0.02 |
| PP_4945 | conserved hypothetical protein gb|AE015451.1|:c5628525-5627689 | -1.35 | **-2.55** | 0.02 |
| PP_0957 | KpsF/GutQ family protein gb|AE015451.1|:c1099563-1098589 | -1.35 | **-2.55** | 0.01 |
| PP_1100 | deoxycytidine triphosphate deaminase gb|AE015451.1|:1257456-1258022 | -1.34 | **-2.54** | 0.01 |
| PP_2006 | hypothetical protein gb|AE015451.1|:c2277453-2275675 | -1.34 | **-2.53** | 0.01 |
| PP_4933 | conserved hypothetical protein gb|AE015451.1|:5613045-5614013 | -1.34 | **-2.53** | 0.01 |
| PP_4938 | glycosyl transferase putative gb|AE015451.1|:c5620495-5619356 | -1.33 | **-2.51** | 0.01 |
| PP_0441 | preprotein translocase SecE subunit gb|AE015451.1|:534102-534470 | -1.32 | **-2.50** | 0.04 |
| PP_5392 | conserved hypothetical protein gb|AE015451.1|:c6147451-6146318 | -1.32 | **-2.50** | 0.01 |
| PP_5391 | hypothetical protein gb|AE015451.1|:c6146308-6145727 | -1.32 | **-2.49** | 0.01 |
| PP_3564 | transcriptional regulator AraC family gb|AE015451.1|:c4043740-4042763 | -1.31 | **-2.49** | 0.02 |
| PP_5027 | D-tyrosyl-tRNA(Tyr) deacylase gb|AE015451.1|:c5729389-5728952 | -1.31 | **-2.47** | 0.01 |
| PP_4852 | transcriptional regulator AraC family gb|AE015451.1|:c5519726-5518938 | -1.30 | **-2.47** | 0.01 |
| PP_4736 | L-lactate dehydrogenase gb|AE015451.1|:5385268-5386413 | -1.30 | **-2.47** | 0.01 |
| PP_4547 | glutamine synthetase putative gb|AE015451.1|:5168381-5169745 | -1.30 | **-2.46** | 0.04 |
| PP_0559 | acetyl-CoA carboxylase biotin carboxyl carrier prote gb|AE015451.1|:c650154-649693 | -1.30 | **-2.45** | 0.01 |
| PP_1073 | glycerol-3-phosphate dehydrogenase gb|AE015451.1|:c1232710-1231166 | -1.29 | **-2.45** | 0.04 |
| PP_1659 | conserved hypothetical protein gb|AE015451.1|:1854985-1856325 | -1.29 | **-2.44** | 0.03 |
| PP_3321 | conserved hypothetical protein gb|AE015451.1|:3757176-3757688 | -1.27 | **-2.41** | 0.02 |
| PP_4636 | beta-ketothiolase gb|AE015451.1|:5259457-5260635 | -1.25 | **-2.39** | 0.03 |
| PP_0453 | ribosomal protein S10 gb|AE015451.1|:550659-550970 | -1.25 | **-2.38** | 0.01 |
| PP_1773 | integration host factor beta subunit gb|AE015451.1|:1981358-1981660 | -1.24 | **-2.36** | 0.01 |
| PP_4192 | succinate dehydrogenase hydrophobic membrane anchor gb|AE015451.1|:c4738740-4738372 | -1.23 | **-2.35** | 0.04 |
| PP_5091 | conserved hypothetical protein gb|AE015451.1|:c5816017-5815598 | -1.22 | **-2.33** | 0.01 |
| nonsymbol | RNA polymerase sigma-54 factor gb|AE015451.1|:c1096058-1094565 | -1.22 | **-2.33** | 0.02 |
| PP_3981 | ISPpu14 transposase Orf3 gb|AE015451.1|:4487111-4488646 | -1.20 | **-2.30** | 0.01 |
| PP_1661 | dehydrogenase subunit putative gb|AE015451.1|:1856818-1859076 | -1.20 | **-2.30** | 0.04 |
| PP_5350 | transcriptional regulator RpiR family gb|AE015451.1|:c6099078-6098212 | -1.19 | **-2.27** | 0.03 |
| PP_3693 | transcriptional regulator MvaT P16 subunit putative gb|AE015451.1|:c4211487-4211122 | -1.18 | **-2.27** | 0.01 |
| PP_1609 | hypothetical protein gb|AE015451.1|:1806092-1806232 | -1.18 | **-2.26** | 0.04 |
| PP_4731 | outer membrane lipoprotein OmlA gb|AE015451.1|:5380697-5381233 | -1.18 | **-2.26** | 0.02 |
| PP_4958 | conserved hypothetical protein gb|AE015451.1|:c5649060-5648521 | -1.17 | **-2.25** | 0.01 |
| PP_3663 | GGDEF domain protein gb|AE015451.1|:c4162052-4160751 | -1.16 | **-2.23** | 0.01 |
| PP_4870 | azurin gb|AE015451.1|:c5537572-5537123 | -1.15 | **-2.22** | 0.04 |
| PP_4193 | succinate dehydrogenase cytochrome b556 subunit gb|AE015451.1|:c4739120-4738734 | -1.15 | **-2.22** | 0.04 |
| PP_4708 | polyribonucleotide nucleotidyltransferase gb|AE015451.1|:c5354114-5352009 | -1.15 | **-2.22** | 0.02 |
| PP_3698 | conserved hypothetical protein gb|AE015451.1|:c4219292-4217793 | -1.15 | **-2.21** | 0.03 |
| PP_1146 | hypothetical protein gb|AE015451.1|:1314805-1315500 | -1.15 | **-2.21** | 0.02 |
| PP_3908 | hypothetical protein gb|AE015451.1|:4418483-4418833 | -1.14 | **-2.21** | 0.03 |
| PP_5396 | ISPpu14 transposase Orf3 gb|AE015451.1|:c6154464-6152929 | -1.14 | **-2.20** | 0.03 |
| PP_4476 | conserved hypothetical protein gb|AE015451.1|:c5085956-5085666 | -1.13 | **-2.19** | 0.05 |
| PP_2313 | conserved hypothetical protein gb|AE015451.1|:2643254-2643691 | -1.13 | **-2.19** | 0.02 |
| PP_4648 | nucleotide methyltransferase putative gb|AE015451.1|:5272852-5273976 | -1.13 | **-2.19** | 0.04 |
| PP_3598 | conserved hypothetical protein gb|AE015451.1|:4087773-4088582 | -1.13 | **-2.18** | 0.02 |
| PP_1714 | peptidyl-prolyl cis-trans isomerase FKBP-type gb|AE015451.1|:1914817-1915569 | -1.13 | **-2.18** | 0.03 |
| PP_4839 | membrane protein putative gb|AE015451.1|:5505171-5506538 | -1.11 | **-2.15** | 0.04 |
| PP_3747 | glycolate oxidase iron-sulfur subunit gb|AE015451.1|:4276603-4277838 | -1.11 | **-2.15** | 0.02 |
| PP_0477 | ribosomal protein S11 gb|AE015451.1|:561868-562257 | -1.10 | **-2.15** | 0.01 |
| PP_0468 | ribosomal protein S8 gb|AE015451.1|:557464-557856 | -1.10 | **-2.14** | 0.02 |
| PP_0476 | ribosomal protein S13 gb|AE015451.1|:561493-561849 | -1.09 | **-2.13** | 0.01 |
| PP_1642 | conserved hypothetical protein gb|AE015451.1|:1837378-1838049 | -1.09 | **-2.12** | 0.03 |
| PP_0450 | ribosomal protein S7 gb|AE015451.1|:546648-547118 | -1.07 | **-2.10** | 0.04 |
| PP_4346 | D-alanine--D-alanine ligase A gb|AE015451.1|:c4938751-4937693 | -1.07 | **-2.10** | 0.05 |
| PP_0339 | pyruvate dehydrogenase E1 component gb|AE015451.1|:c410662-408017 | -1.07 | **-2.09** | 0.04 |
| PP_5088 | primosomal protein N` gb|AE015451.1|:5810664-5812883 | -1.06 | **-2.09** | 0.03 |
| PP_4624 | hydrolase alpha/beta fold family gb|AE015451.1|:c5249813-5248914 | -1.06 | **-2.09** | 0.02 |
| PP_5054 | glutaredoxin gb|AE015451.1|:c5760317-5760063 | -1.06 | **-2.09** | 0.04 |
| PP_5418 | ATP synthase F0 C subunit gb|AE015451.1|:c6180422-6180165 | -1.05 | **-2.07** | 0.03 |
| PP_1212 | conserved domain protein gb|AE015451.1|:1389877-1390098 | -1.05 | **-2.07** | 0.04 |
| PP_3839 | alcohol dehydrogenase zinc-containing gb|AE015451.1|:4362913-4363923 | -1.05 | **-2.07** | 0.02 |
| PP_4714 | conserved hypothetical protein gb|AE015451.1|:c5360836-5360327 | -1.04 | **-2.06** | 0.01 |
| PP_4724 | carbamoyl-phosphate synthase small subunit gb|AE015451.1|:c5373167-5372031 | -1.04 | **-2.06** | 0.02 |
| PP_4652 | membrane protein putative gb|AE015451.1|:5277038-5278258 | -1.04 | **-2.05** | 0.02 |
| PP_1749 | acetyltransferase GNAT family gb|AE015451.1|:c1949651-1947906 | -1.03 | **-2.04** | 0.02 |
| PP_5324 | response regulator gb|AE015451.1|:c6070635-6069745 | -1.03 | **-2.04** | 0.02 |
| PP_3043 | hypothetical protein gb|AE015451.1|:3428087-3428293 | -1.03 | **-2.04** | 0.02 |
| PP_4811 | gamma-glutamyl phosphate reductase gb|AE015451.1|:c5474791-5473520 | -1.02 | **-2.03** | 0.04 |
| PP_2324 | phospho-2-dehydro-3-deoxyheptonate aldolase class I gb|AE015451.1|:c2652414-2651302 | -1.02 | **-2.03** | 0.04 |
| PP_1876 | conserved hypothetical protein gb|AE015451.1|:2099932-2100978 | -1.01 | **-2.02** | 0.05 |
| PP_4788 | conserved hypothetical protein TIGR00043 gb|AE015451.1|:5450121-5450594 | -1.01 | **-2.02** | 0.04 |
| PP_1365 | exodeoxyribonuclease I gb|AE015451.1|:1553925-1555358 | -1.01 | **-2.01** | 0.03 |
| PP_1914 | 3-oxoacyl-(acyl-carrier-protein) reductase gb|AE015451.1|:2157648-2158388 | -1.00 | **-2.01** | 0.02 |
| PP_4283 | transcriptional regulator GntR family gb|AE015451.1|:c4874531-4873776 | -1.00 | **-2.00** | 0.03 |
| PP_5298 | conserved hypothetical protein gb|AE015451.1|:c6046101-6045334 | 1.01 | **2.01** | 0.02 |
| PP_3513 | transcriptional regulator LysR family gb|AE015451.1|:3984714-3985568 | 1.04 | **2.05** | 0.03 |
| PP_0369 | GGDEF domain protein gb|AE015451.1|:447502-448704 | 1.10 | **2.14** | 0.01 |
| PP_4137 | outer membrane siderophore receptor putative gb|AE015451.1|:4673226-4675298 | 1.10 | **2.14** | 0.02 |
| PP_2843 | urease gamma subunit gb|AE015451.1|:3247742-3248044 | 1.11 | **2.15** | 0.04 |
| PP_3951 | 3-oxoadipate CoA-transferase subunit A gb|AE015451.1|:4457362-4458057 | 1.11 | **2.16** | 0.02 |
| PP_3021 | transporter LysE family gb|AE015451.1|:c3408540-3407926 | 1.12 | **2.18** | 0.02 |
| PP_1408 | acyl-transferase gb|AE015451.1|:c1607529-1606642 | 1.13 | **2.18** | 0.04 |
| PP_2093 | response regulator NasT gb|AE015451.1|:c2385874-2385299 | 1.16 | **2.23** | 0.05 |
| PP_4311 | D-amino acid dehydrogenase small subunit putative gb|AE015451.1|:c4904125-4902932 | 1.16 | **2.23** | 0.04 |
| PP_5007 | polyhydroxyalkanoate granule-associated protein GA2 gb|AE015451.1|:c5705363-5704578 | 1.20 | **2.30** | 0.01 |
| PP_0759 | conserved hypothetical protein gb|AE015451.1|:874420-875259 | 1.21 | **2.31** | 0.05 |
| PP_4069 | hypothetical protein gb|AE015451.1|:4594782-4595081 | 1.22 | **2.33** | 0.03 |
| PP_2850 | hypothetical protein gb|AE015451.1|:3252514-3252627 | 1.29 | **2.44** | 0.01 |
| PP_2847 | urease accessory protein UreJ gb|AE015451.1|:3250613-3251158 | 1.29 | **2.44** | 0.02 |
| PP_2689 | endoribonuclease putative gb|AE015451.1|:c3080067-3079696 | 1.29 | **2.45** | 0.04 |
| PP_3902 | hypothetical protein gb|AE015451.1|:c4416483-4416175 | 1.33 | **2.51** | 0.02 |
| PP_5008 | polyhydroxyalkanoate granule-associated protein GA1 gb|AE015451.1|:c5705793-5705374 | 1.34 | **2.54** | 0.01 |
| PP_2687 | conserved hypothetical protein gb|AE015451.1|:c3077849-3076899 | 1.37 | **2.58** | 0.04 |
| PP_2094 | nitrate-binding protein NasS putative gb|AE015451.1|:c2387100-2385886 | 1.37 | **2.58** | 0.03 |
| PP_5233 | ammonium transporter gb|AE015451.1|:c5967683-5966352 | 1.38 | **2.60** | 0.01 |
| PP_2846 | urease accessory protein UreE gb|AE015451.1|:3250091-3250594 | 1.45 | **2.74** | 0.02 |
| PP_1703 | assimilatory nitrate reductase/sulfite reductase put gb|AE015451.1|:1899399-1903472 | 1.85 | **3.61** | 0.01 |
| PP_2092 | nitrate transporter gb|AE015451.1|:c2385138-2383903 | 1.85 | **3.61** | 0.01 |
| PP_2685 | conserved hypothetical protein gb|AE015451.1|:c3076049-3075327 | 1.99 | **3.96** | 0.01 |
| PP_2638 | cellulose synthase operon C protein putative gb|AE015451.1|:3019460-3022978 | 2.09 | **4.26** | 0.00 |
| PP_4841 | branched-chain amino acid ABC transporter periplasmi gb|AE015451.1|:5508393-5509658 | 2.16 | **4.46** | 0.02 |
| PP_2686 | transglutaminase-like superfamily domain protein gb|AE015451.1|:c3076902-3076123 | 2.19 | **4.58** | 0.01 |
| PP_1705 | nitrite reductase gb|AE015451.1|:1903743-1906295 | 2.38 | **5.20** | 0.04 |
| PP_4842 | branched-chain amino acid ABC transporter permease p gb|AE015451.1|:5510246-5511286 | 2.54 | **5.82** | 0.02 |
| PP_5234 | nitrogen regulatory protein P-II gb|AE015451.1|:c5968068-5967730 | 3.61 | **12.22** | 0.01 |

Comparison 2

| ***Locus name*** | ***Gene Name*** | ***Log change*** | ***Fold change*** | ***P-value*** |
| --- | --- | --- | --- | --- |
| PP_0555 | acetoin dehydrogenase alpha subunit gb|AE015451.1|:c645014-644037 | -3.98 | **-15.76** | 0.00 |
| PP_0553 | acetoin dehydrogenase dihydrolipoamide acetyltransfe gb|AE015451.1|:c642984-641878 | -3.65 | **-12.58** | 0.00 |
| PP_0556 | acetoin catabolism protein gb|AE015451.1|:c646092-645037 | -3.20 | **-9.19** | 0.01 |
| PP_3504 | conserved hypothetical protein gb|AE015451.1|:3974810-3975076 | -3.08 | **-8.48** | 0.02 |
| PP_0554 | acetoin dehydrogenase beta subunit gb|AE015451.1|:c644003-642981 | -2.86 | **-7.27** | 0.01 |
| PP_0557 | acetoin catabolism regulatory protein gb|AE015451.1|:646376-648229 | -2.82 | **-7.06** | 0.00 |
| PP_0765 | conserved hypothetical protein gb|AE015451.1|:880484-882370 | -2.79 | **-6.94** | 0.00 |
| PP_4794 | leucyl-tRNA synthetase gb|AE015451.1|:5456267-5458873 | -2.63 | **-6.20** | 0.01 |
| PP_1743 | sodium:solute symporter family protein gb|AE015451.1|:1942832-1944496 | -2.51 | **-5.71** | 0.03 |
| PP_5324 | response regulator gb|AE015451.1|:c6070635-6069745 | -2.50 | **-5.64** | 0.00 |
| PP_0765 | conserved hypothetical protein gb|AE015451.1|:880484-882370 | -2.49 | **-5.62** | 0.00 |
| PP_1659 | conserved hypothetical protein gb|AE015451.1|:1854985-1856325 | -2.28 | **-4.85** | 0.05 |
| PP_2437 | acyl-CoA dehydrogenase putative gb|AE015451.1|:2783545-2784789 | -2.15 | **-4.43** | 0.02 |
| PP_0545 | aldehyde dehydrogenase family protein gb|AE015451.1|:c633438-631918 | -2.13 | **-4.39** | 0.04 |
| PP_2141 | conserved hypothetical protein gb|AE015451.1|:2444126-2444359 | -1.98 | **-3.96** | 0.02 |
| PP_4454 | opine ABC transporter permease protein putative gb|AE015451.1|:c5055386-5054514 | -1.80 | **-3.49** | 0.02 |
| PP_2569 | metabolite MFS transporter MHS family gb|AE015451.1|:2935964-2937640 | -1.62 | **-3.07** | 0.05 |
| PP_0469 | ribosomal protein L6 gb|AE015451.1|:557869-558402 | 1.54 | **2.91** | 0.05 |
| PP_4050 | glycogen synthase gb|AE015451.1|:4564755-4566314 | 1.64 | **3.12** | 0.04 |
| PP_5075 | glutamate synthase small subunit gb|AE015451.1|:c5793321-5791903 | 1.76 | **3.39** | 0.04 |
| PP_1360 | chaperonin 10 kDa gb|AE015451.1|:1549256-1549549 | 1.78 | **3.44** | 0.04 |
| PP_3951 | 3-oxoadipate CoA-transferase subunit A gb|AE015451.1|:4457362-4458057 | 1.87 | **3.67** | 0.04 |
| PP_3105 | hypothetical protein gb|AE015451.1|:3507649-3508344 | 1.96 | **3.89** | 0.05 |
| PP_4573 | ATPase AAA family gb|AE015451.1|:5192819-5193664 | 1.98 | **3.95** | 0.05 |
| PP_1385 | multidrug/solvent RND transporter TtgB gb|AE015451.1|:c1581392-1578240 | 2.01 | **4.02** | 0.01 |
| PP_4550 | long-chain-fatty-acid--CoA ligase gb|AE015451.1|:c5174977-5173289 | 2.07 | **4.19** | 0.04 |
| PP_2074 | transcriptional regulator LysR family gb|AE015451.1|:2359040-2359963 | 2.11 | **4.33** | 0.01 |
| PP_0814 | cytochrome o ubiquinol oxidase subunit III gb|AE015451.1|:953190-953813 | 2.12 | **4.35** | 0.05 |
| PP_2688 | conserved hypothetical protein gb|AE015451.1|:c3079262-3077853 | 2.14 | **4.40** | 0.00 |
| PP_2094 | nitrate-binding protein NasS putative gb|AE015451.1|:c2387100-2385886 | 2.16 | **4.48** | 0.00 |
| PP_4308 | transcriptional regulator AsnC family gb|AE015451.1|:4899601-4900038 | 2.19 | **4.56** | 0.02 |
| PP_0153 | conserved hypothetical protein gb|AE015451.1|:c161965-161750 | 2.23 | **4.69** | 0.04 |
| PP_4054 | conserved hypothetical protein gb|AE015451.1|:4572905-4573189 | 2.26 | **4.80** | 0.01 |
| PP_1794 | hypothetical protein gb|AE015451.1|:c2015484-2014042 | 2.27 | **4.83** | 0.04 |
| PP_0813 | cytochrome o ubiquinol oxidase subunit I gb|AE015451.1|:951168-953186 | 2.31 | **4.95** | 0.03 |
| PP_1795 | hypothetical protein gb|AE015451.1|:c2016389-2015502 | 2.32 | **5.00** | 0.04 |
| PP_5008 | polyhydroxyalkanoate granule-associated protein GA1 gb|AE015451.1|:c5705793-5705374 | 2.62 | **6.14** | 0.00 |
| PP_0811 | cyoups2 protein gb|AE015451.1|:949961-950197 | 2.63 | **6.18** | 0.00 |
| PP_4204 | transcriptional regulator Cro/CI family gb|AE015451.1|:c4750452-4750051 | 2.65 | **6.29** | 0.01 |
| PP_3213 | ABC transporter periplasmic binding component-relate gb|AE015451.1|:c3647060-3646032 | 2.66 | **6.33** | 0.05 |
| PP_1185 | outer membrane protein H1 gb|AE015451.1|:1360133-1360738 | 2.72 | **6.57** | 0.01 |
| PP_2686 | transglutaminase-like superfamily domain protein gb|AE015451.1|:c3076902-3076123 | 2.72 | **6.57** | 0.00 |
| PP_2687 | conserved hypothetical protein gb|AE015451.1|:c3077849-3076899 | 2.82 | **7.07** | 0.00 |
| PP_4841 | branched-chain amino acid ABC transporter periplasmi gb|AE015451.1|:5508393-5509658 | 2.87 | **7.30** | 0.00 |
| PP_2638 | cellulose synthase operon C protein putative gb|AE015451.1|:3019460-3022978 | 2.90 | **7.45** | 0.02 |
| PP_3781 | oxygen-independent Coproporphyrinogen III oxidase fam gb|AE015451.1|:4308593-4310008 | 2.91 | **7.53** | 0.00 |
| PP_1690 | conserved hypothetical protein gb|AE015451.1|:c1883189-1882707 | 3.10 | **8.56** | 0.00 |
| PP_2051 | acetyl-CoA acetyltransferase gb|AE015451.1|:2333066-2334250 | 3.27 | **9.64** | 0.04 |
| PP_2685 | conserved hypothetical protein gb|AE015451.1|:c3076049-3075327 | 3.48 | **11.14** | 0.00 |
| PP_5233 | ammonium transporter gb|AE015451.1|:c5967683-5966352 | 3.77 | **13.67** | 0.00 |
| PP_0367 | conserved hypothetical protein gb|AE015451.1|:444691-445026 | 3.78 | **13.74** | 0.00 |
| PP_4842 | branched-chain amino acid ABC transporter permease p gb|AE015451.1|:5510246-5511286 | 3.99 | **15.91** | 0.00 |
| PP_2048 | acyl-CoA dehydrogenase putative gb|AE015451.1|:2329570-2331420 | 4.31 | **19.87** | 0.00 |
| PP_2047 | 3-hydroxyacyl-CoA dehydrogenase family protein gb|AE015451.1|:2328410-2329648 | 4.39 | **20.91** | 0.00 |
| PP_5234 | nitrogen regulatory protein P-II gb|AE015451.1|:c5968068-5967730 | 4.65 | **25.10** | 0.00 |
| PP_2051 | acetyl-CoA acetyltransferase gb|AE015451.1|:2333066-2334250 | 4.80 | **27.95** | 0.00 |
| PP_2049 | alcohol dehydrogenase iron-containing gb|AE015451.1|:2331454-2332617 | 4.82 | **28.31** | 0.00 |
| PP_2050 | conserved hypothetical protein TIGR00051 gb|AE015451.1|:2332617-2333069 | 4.83 | **28.42** | 0.00 |
