# Supplementary material for: The metabolic response of P. putida KT2442 producing high levels of polyhydroxyalkanoate under single- and multiple-nutrient-limited growth: Highlights from a multi-level omics approach
Source: Microb Cell Fact. 2012 Mar 20;11:34. doi: 10.1186/1475-2859-11-34 (PMC3325844; doi:10.1186/1475-2859-11-34)
Supplement: Additional file 2 — Table S1 Transcriptomic data of genes differentially expressed with a fold change above 2 and below -2 and a P value below 0.05. Comparison 1: CN- vs. C-limited cultures. Comparison 2: N- vs. C- limited cultures. [file 1475-2859-11-34-S2.DOC]

Supplementary Table S2. Proteomics data of proteins that were differentially expressed.

| **SPOT NUMBER** | **ID** | **Enzyme name** | **Fold change** |
| --- | --- | --- | --- |
| **1** | [**gi|26992090**](http://mascot-w2k3/mascot/cgi/protein_view.pl?file=../data/20100511/F079140.dat&hit=1) | **F0F1 ATP synthase subunit alpha** | **91.70** |
| **2** | [**gi|26991521**](http://www.ncbi.nlm.nih.gov/blast/Blast.cgi?ALIGNMENTS=50&ALIGNMENT_VIEW=Pairwise&AUTO_FORMAT=Semiauto&CDD_SEARCH=on&CLIENT=web&COMPOSITION_BASED_STATISTICS=on&DATABASE=nr&DESCRIPTIONS=100&ENTREZ_QUERY=(none)&EXPECT=10&FILTER=L&FORMAT_BLOCK_ON_RESPAGE=None&FORMAT_OBJECT=Alignment&FORMAT_TYPE=HTML&GAPCOSTS=11+1&I_THRESH=0.001&LAYOUT=TwoWindows&MATRIX_NAME=BLOSUM62&NCBI_GI=on&PAGE=Proteins&PROGRAM=blastp&QUERY=MKRRSLIKALTLSASIAAMGLSWSIQAAETIKVGILHSLSGTMAISETSLKDMALMTIEQINAKGGVNGKMLEPVVVDPASNWPLFAEKSRQLLTQDKVAVVFGCWTSVSRKSVLPVFEELNGLLFYPVQYEGEEMSPNVFYTGAAPNQQAIPAVEYLMSEDGGSAKRFFLLGTDYVYPRTTNKILRAFLHSKGVADKDIEEVYTPFGHADYQTIVASIKKFSAGGKTAVISTVNGDSNVPFYKELANQGLKATDVPVVAFSVGEEELRGIDTKPLVGHLAAWNYFESVDNPVNQKFVADWKAYAKAKGLPGADKAVTNDPMEATYVGIHMWAQAVEKAKSTDVDKVREALAGQSFEAPSGFTLTMDKTNHHLHKPVMIGEIQDDGQFSVVWETEQPLRAQPWSPFIPGNDKRPDYAVKGN&SERVICE=plain&SET_DEFAULTS.x=9&SET_DEFAULTS.y=5&SHOW_OVERVIEW=on&WORD_SIZE=3&END_OF_HTTPGET=Yes) | **branched-chain amino acid ABC transporter, periplasmic amino acid-binding protein, putative** | **35.15** |
| **5** | [**gi|26987038**](http://www.ncbi.nlm.nih.gov/blast/Blast.cgi?ALIGNMENTS=50&ALIGNMENT_VIEW=Pairwise&AUTO_FORMAT=Semiauto&CDD_SEARCH=on&CLIENT=web&COMPOSITION_BASED_STATISTICS=on&DATABASE=nr&DESCRIPTIONS=100&ENTREZ_QUERY=(none)&EXPECT=10&FILTER=L&FORMAT_BLOCK_ON_RESPAGE=None&FORMAT_OBJECT=Alignment&FORMAT_TYPE=HTML&GAPCOSTS=11+1&I_THRESH=0.001&LAYOUT=TwoWindows&MATRIX_NAME=BLOSUM62&NCBI_GI=on&PAGE=Proteins&PROGRAM=blastp&QUERY=MKGSPSLLLVALLSAPLLAQAAEPEQCQTVRFSDVGWTDITVTTATTSVVLEALGYKTHTTMISVPVTYKSLATGKDLDVFLGNWMPTMENDIKQYRDAGTVETVRANLENAKYTLAVPQALYDKGLKDFSDIPKFKKELDGKIYGIEPGNDGNRTIQSMIDKNAFGLKDAGFKIVQSSEAGMLSQVDRAQKRGEALVFLGWEPHPMNTRFKMQYLTGGDDFFGPDFGKATVLTNTRKGYTQECSNVGQLLKNLSFELKDESTMMGYVLDDKMKPEAAAKKWIKDNPGKLDAWLAGVTTVDGKPGLEAVKAKLTQ&SERVICE=plain&SET_DEFAULTS.x=9&SET_DEFAULTS.y=5&SHOW_OVERVIEW=on&WORD_SIZE=3&END_OF_HTTPGET=Yes) | **glycine betaine/L-proline ABC transporter, periplasmic binding protein** | **4.88** |
| **6** | [**gi|26991724**](http://mascot-w2k3/mascot/cgi/protein_view.pl?file=../data/20100511/F079148.dat&hit=1) | **nitrogen metabolism transcriptional regulator NtrC** | **6.70** |
| **7** | [**gi|26990756**](http://mascot-w2k3/mascot/cgi/protein_view.pl?file=../data/20100511/F079149.dat&hit=1) | **glycogen debranching protein GlgX** | **4.48** |
| **8** | **gi|26990172** | **hypothetical protein PP_3459** | **5.47** |
| **9** | [**gi|26987893**](http://mascot-w2k3/mascot/cgi/protein_view.pl?file=../data/20100511/F079151.dat&hit=1) | **acetolactate synthase** | **3.46** |
| **10** | [**gi|26988814**](http://mascot-w2k3/mascot/cgi/protein_view.pl?file=../data/20100511/F079152.dat&hit=1) | **OmpF family protein** | **2.60** |
| **11** | [**gi|26987941**](http://mascot-w2k3/mascot/cgi/protein_view.pl?file=../data/20100511/F079153.dat&hit=1) | **outer membrane porin** | **2.68** |
| **12** | [**gi|26991722**](http://mascot-w2k3/mascot/cgi/protein_view.pl?file=../data/20100518/F079246.dat&hit=1) | **glutamine synthetase, type I** | **2.20** |
| **22** | [**gi|26987192**](http://mascot-w2k3/mascot/cgi/protein_view.pl?file=../data/20100605/F079409.dat&hit=1) | **elongation factor G** | **20.03** |
| **23** | [**gi|26991722**](http://mascot-w2k3/mascot/cgi/protein_view.pl?file=../data/20100605/F079410.dat&hit=2) | **glutamine synthetase, type I** | **15.80** |
| **24** | [**gi|26990756**](http://mascot-w2k3/mascot/cgi/protein_view.pl?file=../data/20100605/F079411.dat&hit=1) | **glycogen debranching protein GlgX** | **11.70** |
| **26** | [**gi|26988775**](http://mascot-w2k3/mascot/cgi/protein_view.pl?file=../data/20100605/F079413.dat&hit=1) | **hypothetical protein PP_2050** | **9.38** |
| **27** | [**gi|26991001**](http://mascot-w2k3/mascot/cgi/protein_view.pl?file=../data/20100605/F079414.dat&hit=1) | **hydantoin racemase, putative** | **8.77** |
| **28** | [**gi|26991724**](http://mascot-w2k3/mascot/cgi/protein_view.pl?file=../data/20100511/F079148.dat&hit=1) | **nitrogen metabolism transcriptional regulator NtrC** | **4.87** |
| **29** | [**gi|26988814**](http://mascot-w2k3/mascot/cgi/protein_view.pl?file=../data/20100606/F079416.dat&hit=1) | **OmpF family protein** | **5.43** |
| **30** | [**gi|26987296**](http://mascot-w2k3/mascot/cgi/protein_view.pl?file=../data/20100606/F079417.dat&hit=1) | **acetyl-CoA carboxylase biotin carboxylase subunit** | **8.34** |
| **31** | [**gi|26987458**](http://mascot-w2k3/mascot/cgi/protein_view.pl?file=../data/20100606/F079418.dat&hit=1) | **ribose-phosphate pyrophosphokinase** | **6.55** |
| **32** | [**gi|26989406**](http://mascot-w2k3/mascot/cgi/protein_view.pl?file=../data/20100606/F079420.dat&hit=1) | **hypothetical protein PP_2687** | **5.59** |
| **33** | [**gi|26988830**](http://mascot-w2k3/mascot/cgi/protein_view.pl?file=../data/20100606/F079421.dat&hit=1) | **hypothetical protein PP_2105** | **5.51** |
| **34** | [**gi|26990895**](http://mascot-w2k3/mascot/cgi/protein_view.pl?file=../data/20100606/F079422.dat&hit=1) | **electron-transferring-flavoprotein dehydrogenase** | **4.52** |
| **35** | [**gi|26989812**](http://mascot-w2k3/mascot/cgi/protein_view.pl?file=../data/20100606/F079423.dat&hit=1) | **hypothetical protein PP_3093** | **4.85** |
| **36** | [**gi|26988035**](http://www.ncbi.nlm.nih.gov/blast/Blast.cgi?ALIGNMENTS=50&ALIGNMENT_VIEW=Pairwise&AUTO_FORMAT=Semiauto&CDD_SEARCH=on&CLIENT=web&COMPOSITION_BASED_STATISTICS=on&DATABASE=nr&DESCRIPTIONS=100&ENTREZ_QUERY=(none)&EXPECT=10&FILTER=L&FORMAT_BLOCK_ON_RESPAGE=None&FORMAT_OBJECT=Alignment&FORMAT_TYPE=HTML&GAPCOSTS=11+1&I_THRESH=0.001&LAYOUT=TwoWindows&MATRIX_NAME=BLOSUM62&NCBI_GI=on&PAGE=Proteins&PROGRAM=blastp&QUERY=MSEAIKQPAGPEGIIQMQGVNKWYGQFHVLKDINLNVRQGERIVLCGPSGSGKSTTIRCLNRLEEHQQGRIVVDGVELTNDLKQIEAIRREVGMVFQHFNLFPHLSILENCTLAPMWVRKMPRRKAEEIAMHYLERVRIPEQAHKYPGQLSGGQQQRVAIARALCMKPKIMLFDEPTSALDPEMVKEVLDTMVGLAEDGMTMLCVTHEMGFARTVANRVIFMDKGEIVEQAAPDDFFDRPRSDRTKLFLSQILH&SERVICE=plain&SET_DEFAULTS.x=9&SET_DEFAULTS.y=5&SHOW_OVERVIEW=on&WORD_SIZE=3&END_OF_HTTPGET=Yes) | **general amino acid ABC transporter, ATP-binding protein** | **4.07** |
| **37** | [**gi|26988772**](http://mascot-w2k3/mascot/cgi/protein_view.pl?file=../data/20100531/F079299.dat&hit=1) | **3-hydroxyacyl-CoA dehydrogenase family protein** | **3.97** |
| **38** | [**gi|26988860**](http://mascot-w2k3/mascot/cgi/protein_view.pl?file=../data/20100531/F079300.dat&hit=1) | **multifunctional fatty acid oxidation complex subunit alpha** | **3.06** |
| **39** | **gi|26989257** | **hypothetical protein PP_2537** | **3.17** |
| **40** | **gi|26988861** | **3-ketoacyl-CoA thiolase** | **2.95** |
| **41** | [**gi|26990883**](http://mascot-w2k3/mascot/cgi/protein_view.pl?file=../data/20100601/F079312.dat&hit=1) | **succinate dehydrogenase flavoprotein subunit** | **3.93** |
| **43** | [**gi|26988860**](http://mascot-w2k3/mascot/cgi/protein_view.pl?file=../data/20100601/F079315.dat&hit=1) | **multifunctional fatty acid oxidation complex subunit alpha** | **3.06** |
| **44** | [**gi|26990817**](http://www.ncbi.nlm.nih.gov/blast/Blast.cgi?ALIGNMENTS=50&ALIGNMENT_VIEW=Pairwise&AUTO_FORMAT=Semiauto&CDD_SEARCH=on&CLIENT=web&COMPOSITION_BASED_STATISTICS=on&DATABASE=nr&DESCRIPTIONS=100&ENTREZ_QUERY=(none)&EXPECT=10&FILTER=L&FORMAT_BLOCK_ON_RESPAGE=None&FORMAT_OBJECT=Alignment&FORMAT_TYPE=HTML&GAPCOSTS=11+1&I_THRESH=0.001&LAYOUT=TwoWindows&MATRIX_NAME=BLOSUM62&NCBI_GI=on&PAGE=Proteins&PROGRAM=blastp&QUERY=MTITSFGPANRIARSAETHPLTWRLRDDGEPVWLAEYESKNGYAAARKALAQMSADDIVQSVKDSGLKGRGGAGFPTGVKWGLMPKDESMNIRYLLCNADEMEPNTWKDRMLMEQQPHLLVEGMLISARALKAYRGYIFLRGEYTTAAKNLNRAIDEAKAAGLLGKNILGSGFDFELFVHTGAGRYICGEETALINSLEGRRANPRSKPPFPAAVGVWGKPTCVNNVETLCNVPAIVANGNDWYKSLAREGSEDHGTKLMGFSGKVKNPGLWELPFGVTARELFEDYAGGMRDGFKLKCWQPGGAGTGFLLPEHLDAQMYAGGIAKVGTRMGTGLAMAVDDSINMVSLLRNMEEFFARESCGWCTPCRDGLPWSVKMLRALENGQGRAEDIETLLGLVNFLGPGRTFCAHAPGAVEPLGSAIKYFRSEFEAGVAPESAATLRPDLAKPIVVGA&SERVICE=plain&SET_DEFAULTS.x=9&SET_DEFAULTS.y=5&SHOW_OVERVIEW=on&WORD_SIZE=3&END_OF_HTTPGET=Yes) | **NADH dehydrogenase I subunit F** | **2.89** |
| **45** | **gi|26990754** | **malto-oligosyltrehalose synthase** | **2.56** |
| **46** | **gi|26988807** | **phosphoenolpyruvate synthase** | **2.35** |
| **13** | [**gi|26991567**](http://mascot-w2k3/mascot/cgi/protein_view.pl?file=../data/20100605/F079399.dat&hit=1) | **adenylosuccinate synthetase** | **0.23** |
| **14** | [**gi|26990716**](http://mascot-w2k3/mascot/cgi/protein_view.pl?file=../data/20100605/F079400.dat&hit=2) | **isocitrate dehydrogenase, NADP-dependent** | **0.20** |
| **15** | [**gi|26987293**](http://mascot-w2k3/mascot/cgi/protein_view.pl?file=../data/20100605/F079401.dat&hit=1) | **pyruvate dehydrogenase (acetyl-transferring)** | **0.25** |
| **16** | [**gi|26987292**](http://mascot-w2k3/mascot/cgi/protein_view.pl?file=../data/20100605/F079402.dat&hit=1) | **acetoin dehydrogenase, beta subunit** | **0.18** |
| **17** | [**gi|26987291**](http://mascot-w2k3/mascot/cgi/protein_view.pl?file=../data/20100605/F079403.dat&hit=1) | **branched-chain alpha-keto acid dehydrogenase subunit E2** | **0.27** |
| **18** | [**gi|26989108**](http://mascot-w2k3/mascot/cgi/protein_view.pl?file=../data/20100605/F079405.dat&hit=1) | **branched chain amino acid ABC transporter** | **0.36** |
| **19** | [**gi|26987716**](http://mascot-w2k3/mascot/cgi/protein_view.pl?file=../data/20100605/F079406.dat&hit=1) | **leucyl aminopeptidase** | **0.44** |
| **47** | [**gi|26987024**](http://mascot-w2k3/mascot/cgi/protein_view.pl?file=../data/20100601/F079319.dat&hit=1) | **amino acid ABC transporter, periplasmic amino acid-binding protein** | **0.03** |
| **48** | [**gi|26988458**](http://mascot-w2k3/mascot/cgi/protein_view.pl?file=../data/20100601/F079321.dat&hit=1) | **ABC transporter, periplasmic binding protein** | **0.04** |
| **49** | [**gi|26991547**](http://mascot-w2k3/mascot/cgi/protein_view.pl?file=../data/20100601/F079327.dat&hit=1) | **extracellular ligand-binding receptor** | **0.06** |
| **50** | [**gi|26987651**](http://mascot-w2k3/mascot/cgi/protein_view.pl?file=../data/20100601/F079328.dat&hit=1) | **superoxide dismutase** | **0.05** |
| **51** | [**gi|26992051**](http://mascot-w2k3/mascot/cgi/protein_view.pl?file=../data/20100601/F079329.dat&hit=1) | **LysR family transcriptional regulator** | **0.08** |
| **52** | [**gi|26991377**](http://mascot-w2k3/mascot/cgi/protein_view.pl?file=../data/20100601/F079330.dat&hit=1) | **C4-type zinc finger DksA/TraR family protein** | **0.09** |
| **54** | **gi|26991067** | **flagellin FliC** | **0.17** |
| **55** | [**gi|26988588**](http://mascot-w2k3/mascot/cgi/protein_view.pl?file=../data/20100605/F079366.dat&hit=1) | **elongation factor P** | **0.12** |
| **56** | [**gi|26988743**](http://mascot-w2k3/mascot/cgi/protein_view.pl?file=../data/20100605/F079367.dat&hit=1) | **BNR domain-containing protein** | **0.13** |
| **57** | [**gi|26987098**](http://mascot-w2k3/mascot/cgi/protein_view.pl?file=../data/20100605/F079368.dat&hit=1) | **malate synthase G** | **0.11** |
| **58** | [**gi|26988076**](http://mascot-w2k3/mascot/cgi/protein_view.pl?file=../data/20100605/F079369.dat&hit=1) | **cell division protein FtsZ** | **0.14** |
| **59** | [**gi|26986857**](http://mascot-w2k3/mascot/cgi/protein_view.pl?file=../data/20100605/F079370.dat&hit=1) | **metal ABC transporter periplasmic-binding protein** | **0.13** |
| **60** | [**gi|26991413**](http://mascot-w2k3/mascot/cgi/protein_view.pl?file=../data/20100605/F079371.dat&hit=1) | **ferric uptake regulator, Fur family** | **0.16** |
| **62** | [**gi|26987621**](http://mascot-w2k3/mascot/cgi/protein_view.pl?file=../data/20100605/F079373.dat&hit=1) | **dipeptide ABC transporter, periplasmic peptide-binding protein** | **0.20** |
| **63** | [**gi|26987283**](http://mascot-w2k3/mascot/cgi/protein_view.pl?file=../data/20100605/F079374.dat&hit=1) | **aldehyde dehydrogenase family protein** | **0.17** |
| **64** | [**gi|26990223**](http://mascot-w2k3/mascot/cgi/protein_view.pl?file=../data/20100605/F079375.dat&hit=1) | **branched-chain amino acid aminotransferase** | **0.32** |
| **65** | [**gi|26991067**](http://mascot-w2k3/mascot/cgi/protein_view.pl?file=../data/20100605/F079376.dat&hit=1) | **flagellin FliC** | **0.18** |
| **67** | [**gi|26988032**](http://www.ncbi.nlm.nih.gov/blast/Blast.cgi?ALIGNMENTS=50&ALIGNMENT_VIEW=Pairwise&AUTO_FORMAT=Semiauto&CDD_SEARCH=on&CLIENT=web&COMPOSITION_BASED_STATISTICS=on&DATABASE=nr&DESCRIPTIONS=100&ENTREZ_QUERY=(none)&EXPECT=10&FILTER=L&FORMAT_BLOCK_ON_RESPAGE=None&FORMAT_OBJECT=Alignment&FORMAT_TYPE=HTML&GAPCOSTS=11+1&I_THRESH=0.001&LAYOUT=TwoWindows&MATRIX_NAME=BLOSUM62&NCBI_GI=on&PAGE=Proteins&PROGRAM=blastp&QUERY=MKMLKTTLAVLTAAAALGAVSTAQAGATLDAVKKKGFVQCGVSDGLPGFSVPDAQGKIVGIDADVCRAVAAAVFGDATKVKFSQLNAKERFTALQSGEVDVLSRNTTWTSSRDAGMGLVFAGVTYYDGVGFLVNKKLGVSSAKELDGATICIQAGTTTELNVSDFFRANGLKYTPITFDTSDESAKSLESGRCDVLTSDKSQLFAQRSKLAAPTEYVVLPETISKEPLGPVVRKGDEEWFSIVKWTLFAMLNAEEAGITSKNVEAEAKATKNPDVARLLGADGEYGKDLKLPKDWVVQIVKQVGNYGEVFEKNLGQSTDLKIDRGMNALWNNGGIQYAPPVR&SERVICE=plain&SET_DEFAULTS.x=9&SET_DEFAULTS.y=5&SHOW_OVERVIEW=on&WORD_SIZE=3&END_OF_HTTPGET=Yes) | **general amino acid ABC transporter, periplasmic binding protein** | **0.16** |
| **68** | [**gi|26990810**](http://mascot-w2k3/mascot/cgi/protein_view.pl?file=../data/20100605/F079379.dat&hit=1) | **isocitrate lyase** | **0.24** |
| **69** | [**gi|26990740**](http://mascot-w2k3/mascot/cgi/protein_view.pl?file=../data/20100605/F079379.dat&hit=2) | **dihydropyrimidine dehydrogenase** | **0.29** |
| **70** | [**gi|26990737**](http://mascot-w2k3/mascot/cgi/protein_view.pl?file=../data/20100605/F079381.dat&hit=1) | **allantoate amidohydrolase** | **0.22** |
| **71** | [**gi|26987501**](http://mascot-w2k3/mascot/cgi/protein_view.pl?file=../data/20100605/F079382.dat&hit=1) | **hypothetical protein PP_0765** | **0.22** |
|  |  |  |  |
| **72** | [**gi|26988094**](http://mascot-w2k3/mascot/cgi/protein_view.pl?file=../data/20100605/F079383.dat&hit=1) | **co-chaperonin GroES** | **0.36** |
| **73** | [**gi|26990893**](http://www.ncbi.nlm.nih.gov/blast/Blast.cgi?ALIGNMENTS=50&ALIGNMENT_VIEW=Pairwise&AUTO_FORMAT=Semiauto&CDD_SEARCH=on&CLIENT=web&COMPOSITION_BASED_STATISTICS=on&DATABASE=nr&DESCRIPTIONS=100&ENTREZ_QUERY=(none)&EXPECT=10&FILTER=L&FORMAT_BLOCK_ON_RESPAGE=None&FORMAT_OBJECT=Alignment&FORMAT_TYPE=HTML&GAPCOSTS=11+1&I_THRESH=0.001&LAYOUT=TwoWindows&MATRIX_NAME=BLOSUM62&NCBI_GI=on&PAGE=Proteins&PROGRAM=blastp&QUERY=MTILVVAEYEAGAVAPATLNTVAAAAKIGGDVHVLVAGQNVGGVAESAAKIAGVAKVLVADNAAYAHVLPENVAPLIVELAKGYSHVLAPATTNGKNILPRVAALLDVDQISEIISVESADTFKRPIYAGNAIATVQSSAAIKVITVRTTGFDAVAAEGGSAAVEAVGAAHNAGISAFVGEELAKSDRPELTAAKIVVSGGRGMGNGDNFKHLYSLADKLGAAVGASRAAVDAGFVPNDMQVGQTGKIVAPQLYIAVGISGAIQHLAGMKDSKVIVAINKDEEAPIFQVADYGLVADLFEAVPELEKLV&SERVICE=plain&SET_DEFAULTS.x=9&SET_DEFAULTS.y=5&SHOW_OVERVIEW=on&WORD_SIZE=3&END_OF_HTTPGET=Yes) | **electron transfer flavoprotein, alpha subunit** | **0.27** |
| **74** | [**gi|26987098**](http://mascot-w2k3/mascot/cgi/protein_view.pl?file=../data/20100605/F079386.dat&hit=1) | **malate synthase G** | **0.25** |
| **76** | [**gi|26991857**](http://mascot-w2k3/mascot/cgi/protein_view.pl?file=../data/20100605/F079388.dat&hit=1) | **putrescine ABC transporter, periplasmic putrescine-binding protein** | **0.33** |
| **77** | [**gi|26991502**](http://mascot-w2k3/mascot/cgi/protein_view.pl?file=../data/20100605/F079389.dat&hit=1) | **bifunctional phosphoribosylaminoimidazolecarboxamide formyltransferase/IMP cyclohydrolase** | **0.22** |
| **78** | **gi|26987250** | **hypothetical protein PP_0512** | **0.35** |
| **79** | [**gi|26987499**](http://mascot-w2k3/mascot/cgi/protein_view.pl?file=../data/20100605/F079391.dat&hit=1) | **acyl-CoA synthetase** | **0.27** |
| **80** | [**gi|26987501**](http://mascot-w2k3/mascot/cgi/protein_view.pl?file=../data/20100605/F079392.dat&hit=1) | **hypothetical protein PP_0765** | **0.37** |
| **81** | [**gi|26987585**](http://mascot-w2k3/mascot/cgi/protein_view.pl?file=../data/20100605/F079393.dat&hit=1) | **nucleoside-diphosphate kinase** | **0.30** |
| **83** | [**gi|26987407**](http://mascot-w2k3/mascot/cgi/protein_view.pl?file=../data/20100605/F079395.dat&hit=1) | **serine hydroxymethyltransferase** | **0.28** |
| **84** | [**gi|161378124**](http://mascot-w2k3/mascot/cgi/protein_view.pl?file=../data/20100605/F079396.dat&hit=1) | **flagellum-specific ATP synthase** | **0.44** |
| **85** | [**gi|26987578**](http://mascot-w2k3/mascot/cgi/protein_view.pl?file=../data/20100605/F079397.dat&hit=1) | **cysteine desulfurase IscS** | **0.35** |
| **86** | [**gi|26990716**](http://mascot-w2k3/mascot/cgi/protein_view.pl?file=../data/20100605/F079398.dat&hit=1) | **isocitrate dehydrogenase, NADP-dependent** | **0.48** |
| **20** | [**gi|26987193**](http://www.ncbi.nlm.nih.gov/blast/Blast.cgi?ALIGNMENTS=50&ALIGNMENT_VIEW=Pairwise&AUTO_FORMAT=Semiauto&CDD_SEARCH=on&CLIENT=web&COMPOSITION_BASED_STATISTICS=on&DATABASE=nr&DESCRIPTIONS=100&ENTREZ_QUERY=(none)&EXPECT=10&FILTER=L&FORMAT_BLOCK_ON_RESPAGE=None&FORMAT_OBJECT=Alignment&FORMAT_TYPE=HTML&GAPCOSTS=11+1&I_THRESH=0.001&LAYOUT=TwoWindows&MATRIX_NAME=BLOSUM62&NCBI_GI=on&PAGE=Proteins&PROGRAM=blastp&QUERY=MAKEKFDRSLPHVNVGTIGHVDHGKTTLTAALTRVCSEVFGSAVVEFDKIDSAPEEKARGITINTAHVEYNSNIRHYAHVDCPGHADYVKNMITGAAQMDGAILVCSAADGPMPQTREHILLSRQVGVPYIVVFLNKADLVDDAELLELVEMEVRDLLSTYDFPGDDTPIIIGSARMALEGKDDNEMGTTAVKKLVETLDAYIPEPVRAIDQPFLMPIEDVFSISGRGTVVTGRIERGIVRVQDPLEIVGLRDTTTTTCTGVEMFRKLLDEGRAGENCGVLLRGTKRDDVERGQVLVKPGSVKPHTKFTAEVYVLSKEEGGRHTPFFKGYRPQFYFRTTDVTGNCELPEGVEMVMPGDNIQMTVTLIKTIAMEDGLRFAIREGGRTVGAGVVAKIIE&SERVICE=plain&SET_DEFAULTS.x=9&SET_DEFAULTS.y=5&SHOW_OVERVIEW=on&WORD_SIZE=3&END_OF_HTTPGET=Yes) | **elongation factor Tu** | **Novo C/N-C (UP)** |
| **21** | [**gi|26991910**](http://www.ncbi.nlm.nih.gov/blast/Blast.cgi?ALIGNMENTS=50&ALIGNMENT_VIEW=Pairwise&AUTO_FORMAT=Semiauto&CDD_SEARCH=on&CLIENT=web&COMPOSITION_BASED_STATISTICS=on&DATABASE=nr&DESCRIPTIONS=100&ENTREZ_QUERY=(none)&EXPECT=10&FILTER=L&FORMAT_BLOCK_ON_RESPAGE=None&FORMAT_OBJECT=Alignment&FORMAT_TYPE=HTML&GAPCOSTS=11+1&I_THRESH=0.001&LAYOUT=TwoWindows&MATRIX_NAME=BLOSUM62&NCBI_GI=on&PAGE=Proteins&PROGRAM=blastp&QUERY=MKLVTAIIKPFKLDDVRESLSEIGVQGITVTEVKGFGRQKGHTELYRGAEYVVDFLPKVKIDVAIDDKDLDRVIEAITKAANTGKIGDGKIFVVNLEQAIRIRTGETDTDAI&SERVICE=plain&SET_DEFAULTS.x=9&SET_DEFAULTS.y=5&SHOW_OVERVIEW=on&WORD_SIZE=3&END_OF_HTTPGET=Yes) | **nitrogen regulatory protein P-II** | **Novo C/N-C (UP)** |
| **87** | [**gi|26988772**](http://www.ncbi.nlm.nih.gov/blast/Blast.cgi?ALIGNMENTS=50&ALIGNMENT_VIEW=Pairwise&AUTO_FORMAT=Semiauto&CDD_SEARCH=on&CLIENT=web&COMPOSITION_BASED_STATISTICS=on&DATABASE=nr&DESCRIPTIONS=100&ENTREZ_QUERY=(none)&EXPECT=10&FILTER=L&FORMAT_BLOCK_ON_RESPAGE=None&FORMAT_OBJECT=Alignment&FORMAT_TYPE=HTML&GAPCOSTS=11+1&I_THRESH=0.001&LAYOUT=TwoWindows&MATRIX_NAME=BLOSUM62&NCBI_GI=on&PAGE=Proteins&PROGRAM=blastp&QUERY=MNTPFPINQVAVIGAGTMGRGIVISLANAGLSVLWLDCNAAALEAGLGMVSQAWAQQVDKQRITQAQADACLARVQAVDGYPALAEADLVIEAVYENLALKQEIFCALDAHLKPRAILASNTSALDIDAIAAVTQRPSQVLGLHFFSPAHVMKLLEIVRGTHTDQKVLDAAKALGERMGKVAIVAGNCPGFIGNRMLRSYVGEARKLLLEGALPHQVDAVLQQFGFAMGPFRMYDVVGIDLEWRARQLAGQGMHDPLVQVDNALCELGRLGQKTGQGYYRYAPGSRQAEHDPQVDALVLQVSQNLGYRRRGISAEEILERCLLALVNEGAKVLQEGIAASSGDIDQVWLHGYGFPAATGGPMRWADEQGAPFILARLEYLQGVLGEHWRPAGLLYSLVAGGKRFEPRGEVQA&SERVICE=plain&SET_DEFAULTS.x=9&SET_DEFAULTS.y=5&SHOW_OVERVIEW=on&WORD_SIZE=3&END_OF_HTTPGET=Yes) | **3-hydroxyacyl-CoA dehydrogenase family protein** | **Novo N-C (UP)** |
| **88** | [**gi|26987920**](http://mascot-w2k3/mascot/cgi/protein_view.pl?file=../data/20100531/F079302.dat&hit=1) | **outer membrane protein H1** | **Novo N-C (UP)** |
| **89** | [**gi|26988776**](http://www.ncbi.nlm.nih.gov/blast/Blast.cgi?ALIGNMENTS=50&ALIGNMENT_VIEW=Pairwise&AUTO_FORMAT=Semiauto&CDD_SEARCH=on&CLIENT=web&COMPOSITION_BASED_STATISTICS=on&DATABASE=nr&DESCRIPTIONS=100&ENTREZ_QUERY=(none)&EXPECT=10&FILTER=L&FORMAT_BLOCK_ON_RESPAGE=None&FORMAT_OBJECT=Alignment&FORMAT_TYPE=HTML&GAPCOSTS=11+1&I_THRESH=0.001&LAYOUT=TwoWindows&MATRIX_NAME=BLOSUM62&NCBI_GI=on&PAGE=Proteins&PROGRAM=blastp&QUERY=MKDAVIVATARTPIGKAMRGAFNDLKTPSMTAVAIRAAVERAGIEPAQVDDLVLGTAMQSGTAAINPGRLSALAAGLPQSVSGQTVDRQCASGLMAIATAAKQIMVDGMQVTIGAGQEQISLVQQVHNQLASEAYDPAVLRMSEHAYMPMLQTAERVARRYGISREAQDVYALQSQQRTAAAQAAGLFAAEIVPVIARKKVVDKLTGVVSHEEVRLTQDEGNRPATTLADLQGLKPVVEGGCVTAGNASQLSDGASACVLMEGALAARSGIAALGLYRGIAVAGLAPEEMGIGPVLAVPKLLRQQGLTVDDIGLWELNEAFACQVLYCAQQLQIDPAKLNVNGGAIAIGHPYGMSGARMVGHALLEGKRRKVKYVVVTMCVGGGMGAAGLFEVL&SERVICE=plain&SET_DEFAULTS.x=9&SET_DEFAULTS.y=5&SHOW_OVERVIEW=on&WORD_SIZE=3&END_OF_HTTPGET=Yes) | **acetyl-CoA acetyltransferase** | **Novo N-C (UP)** |
| **90** | **gi|26992090** | **F0F1 ATP synthase subunit alpha** | **Novo N-C (UP)** |
| **91** | [**gi|26990877**](http://mascot-w2k3/mascot/cgi/protein_view.pl?file=../data/20100531/F079305.dat&hit=1) | **succinyl-CoA synthetase subunit alpha** | **Novo N-C (UP)** |
| **92** | [**gi|26989024**](http://mascot-w2k3/mascot/cgi/protein_view.pl?file=../data/20100601/F079306.dat&hit=1) | **ATP-dependent Clp protease proteolytic subunit** | **Novo N-C (UP)** |
| **93** | [**gi|26991544**](http://www.ncbi.nlm.nih.gov/blast/Blast.cgi?ALIGNMENTS=50&ALIGNMENT_VIEW=Pairwise&AUTO_FORMAT=Semiauto&CDD_SEARCH=on&CLIENT=web&COMPOSITION_BASED_STATISTICS=on&DATABASE=nr&DESCRIPTIONS=100&ENTREZ_QUERY=(none)&EXPECT=10&FILTER=L&FORMAT_BLOCK_ON_RESPAGE=None&FORMAT_OBJECT=Alignment&FORMAT_TYPE=HTML&GAPCOSTS=11+1&I_THRESH=0.001&LAYOUT=TwoWindows&MATRIX_NAME=BLOSUM62&NCBI_GI=on&PAGE=Proteins&PROGRAM=blastp&QUERY=MSDDIILSVDNLMMQFGGIKALSDVSLKVRRNQIFALIGPNGAGKTTVFNCLTGFYKASGGRIELNVRGSHTNVIQLLGERFQAADFVSPARFANRMYYKMFGGTHLVNRAGLARTFQNIRLFKEMSVVENLLVAQHMWVNRNLLAGVLNTKAYRKAESDALDHAFYWLEVVDLVDCANRLAGELSYGQQRRLEIARAMCTRPKIICLDEPAAGLNPQETEALSRMIRVLRDEHDITVVLIEHDMGMVMSISDHIVVLDHGNVIAEGAPQDIRHNPTVIAAYLGADEEELV&SERVICE=plain&SET_DEFAULTS.x=9&SET_DEFAULTS.y=5&SHOW_OVERVIEW=on&WORD_SIZE=3&END_OF_HTTPGET=Yes) | **branched chain amino acid ABC transporter ATP-binding protein** | **Novo N-C (UP)** |
| **94** | **gi|26987502** | **hypothetical protein PP_0766** | **Novo N-C (DOWN)** |
| **95** | [**gi|26987877**](http://mascot-w2k3/mascot/cgi/protein_view.pl?file=../data/20100601/F079323.dat&hit=1) | **extracellular ligand-binding receptor** | **Novo N-C (DOWN)** |
| **96** | [**gi|26987807**](http://mascot-w2k3/mascot/cgi/protein_view.pl?file=../data/20100601/F079324.dat&hit=1) | **amino acid ABC transporter, periplasmic amino acid-binding protein** | **Novo N-C (DOWN)** |
|  |  |  | **Novo N-C (DOWN)** |
| **98** | [**gi|26987292**](http://mascot-w2k3/mascot/cgi/protein_view.pl?file=../data/20100605/F079402.dat&hit=1) | **acetoin dehydrogenase, beta subunit** | **Novo N-C (DOWN)** |
| **99** | [**gi|26987291**](http://mascot-w2k3/mascot/cgi/protein_view.pl?file=../data/20100605/F079403.dat&hit=1) | **branched-chain alpha-keto acid dehydrogenase subunit E2** | **Novo N-C (DOWN)** |
