# Supplementary material for: The metabolic response of P. putida KT2442 producing high levels of polyhydroxyalkanoate under single- and multiple-nutrient-limited growth: Highlights from a multi-level omics approach
Source: Microb Cell Fact. 2012 Mar 20;11:34. doi: 10.1186/1475-2859-11-34 (PMC3325844; doi:10.1186/1475-2859-11-34)
Supplement: Additional file 3 — Table S2 Proteomics data of proteins that were differentially expressed. [file 1475-2859-11-34-S3.DOC]

Supplementary Table S3. Transcriptomic data of genes differentially expressed with a fold change above 3 and a *P* value below 0.02. Nitrogen- vs. dual-nutrient-limited cultures.

| **Locus Name** | **Gene Name** | Log change | Fold change | *p*-value |
| --- | --- | --- | --- | --- |
| PP_0389 | ribosomal protein S21 gb|AE015451.1|:c475503-475288 | 1.85 | **3.62** | 0.00 |
| PP_1099 | cold-shock domain family protein gb|AE015451.1|:c1257128-1256919 | 1.89 | **3.72** | 0.02 |
| PP_0008 | ribonuclease P protein component gb|AE015451.1|:c8798-8394 | 1.99 | **3.98** | 0.02 |
| PP_1083 | bacterioferritin-associated ferredoxin putative gb|AE015451.1|:c1242882-1242664 | 2.03 | **4.09** | 0.02 |
| PP_2048 | acyl-CoA dehydrogenase putative gb|AE015451.1|:2329570-2331420 | 3.32 | **10.01** | 0.00 |
| PP_2051 | acetyl-CoA acetyltransferase gb|AE015451.1|:2333066-2334250 | 3.37 | **10.36** | 0.01 |
| PP_2050 | conserved hypothetical protein TIGR00051 gb|AE015451.1|:2332617-2333069 | 3.64 | **12.45** | 0.00 |
| PP_2049 | alcohol dehydrogenase iron-containing gb|AE015451.1|:2331454-2332617 | 4.00 | **16.05** | 0.00 |
| PP_2047 | 3-hydroxyacyl-CoA dehydrogenase family protein gb|AE015451.1|:2328410-2329648 | 4.69 | **25.84** | 0.00 |
