# Supplementary figures and images for: The metabolic response of P. putida KT2442 producing high levels of polyhydroxyalkanoate under single- and multiple-nutrient-limited growth: Highlights from a multi-level omics approach
Source: Microb Cell Fact. 2012 Mar 20;11:34. doi: 10.1186/1475-2859-11-34 (PMC3325844; doi:10.1186/1475-2859-11-34)

**
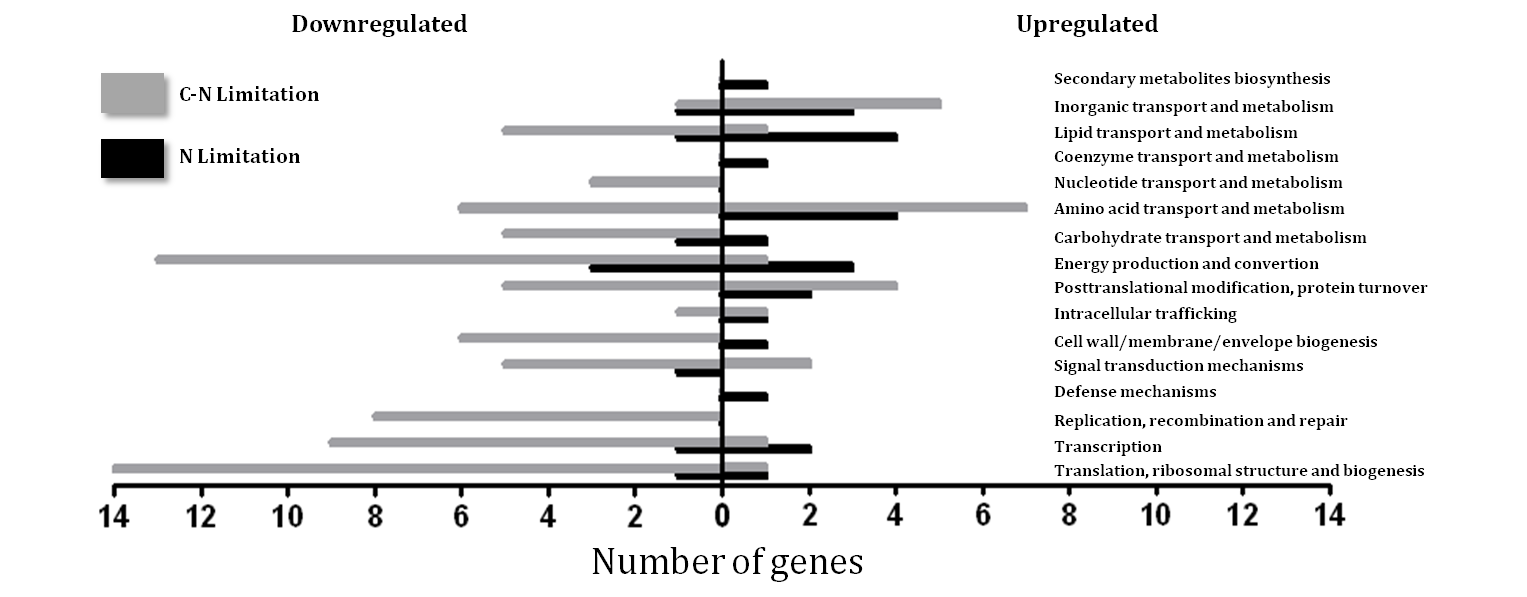
**

Supplement: Additional file 4 — Table S3 Transcriptomic data of genes differentially expressed with a fold change above 3 and a P value below 0.02. Nitrogen- vs. dual-nutrient-limited cultures. [file 1475-2859-11-34-S4.DOC]
